# Exploring novel isatin derivatives as SARS-CoV-2 3CL^pro^ inhibitors

**DOI:** 10.1080/14756366.2026.2707859

**Published:** 2026-09-10

**Authors:** Jian Song, Cai Shi, Boning Yang, Jingxiang Yang, Caifei Huang, Jiaqi Liu, Shuwen Wu, Shuyuan Mo, Jian Yang

**Affiliations:** ^a^ Department of Pharmacy, Renmin Hospital of Wuhan University, Wuhan, China; ^b^ Division of Pharmacy and Optometry, University of Manchester, Manchester, UK; ^c^ Department of Pharmacy, Wuhan Children’s Hospital, Tongji Medical College, Huazhong University of Science & Technology, Wuhan, China; ^d^ The College of Life Sciences, State Key Laboratory of Virology, Modern Virology Research Center, Wuhan University, Wuhan, China

**Keywords:** SARS-CoV-2 3CLpro inhibitors, isatin derivatives, molecular docking, molecular dynamics

## Abstract

A series of novel isatin derivatives were synthesised by incorporating phenyl, biphenyl, naphthyl and indolyl moieties into the N-1 position and screened in vitro against SARS-CoV-2 3CL^pro^, respectively. These isatin compounds with halogen substitution at the isatin ring and trifluoromethylbenzyl substitution at N-1 position were strong inhibitors of SARS-CoV-2 3CL^pro^. Furthermore, introduction of electron withdrawing groups at the C-5 and C-7 of isatin ring, such as bromo group, might be more favourable for the inhibition activity. The most potent compound 34 demonstrated an IC_50_ of 0.363 ± 0.045 µM against SARS-CoV-2 3CL^pro^. Additionally, a jump dilution assay, surface plasmon resonance (SPR) spectroscopy and in silico analysis were used to measure the binding of compound **34** to SARS-CoV-2 3CL^pro^ respectively, supporting the obtained in vitro findings. Moreover, compound **34** inhibited viral cell proliferation with an IC_50_ of 0.404 ± 0.021 μM (selectivity index (SI) =170). Therefore, it is worth to further investigate compound **34** as a SARS-CoV-2 3CL^pro^ inhibitor.

## Introduction

Since the World Health Organisation (WHO) declared COVID-19 a pandemic in 2019, the rapid spread of SARS-CoV-2 has resulted in more than 620 million confirmed cases and the deaths of millions of people, making it one of the most catastrophic global health disasters in human history^1^^,^^2^. Despite the deleterious consequences of COVID-19 and the huge efforts expended on finding efficient therapies, no single drug has proven effective in the treatment of COVID-19 patients, and the currently adopted therapeutic strategies are either supportive or preventative^3^^,^^4^.

To date, several potential SARS-CoV-2 3CL^pro^ inhibitors have been reported from compound library screening^5^, rational design^5–7^, and testing of ingredients from traditional Chinese medicine^8^. The chemical structures of the experimentally identified SARS-CoV-2 3CL^pro^ inhibitors are diverse, including a-ketoamide analogues^6^, peptidomimetics compounds^5^^,^^7^, baicalein and its derivatives^8^, and several repurposed approved drugs and drug candidates^5^. However, there are now only a few SARS-CoV-2 virus medicines available, despite the wide spread approval of vaccination.

**Figure 1.**
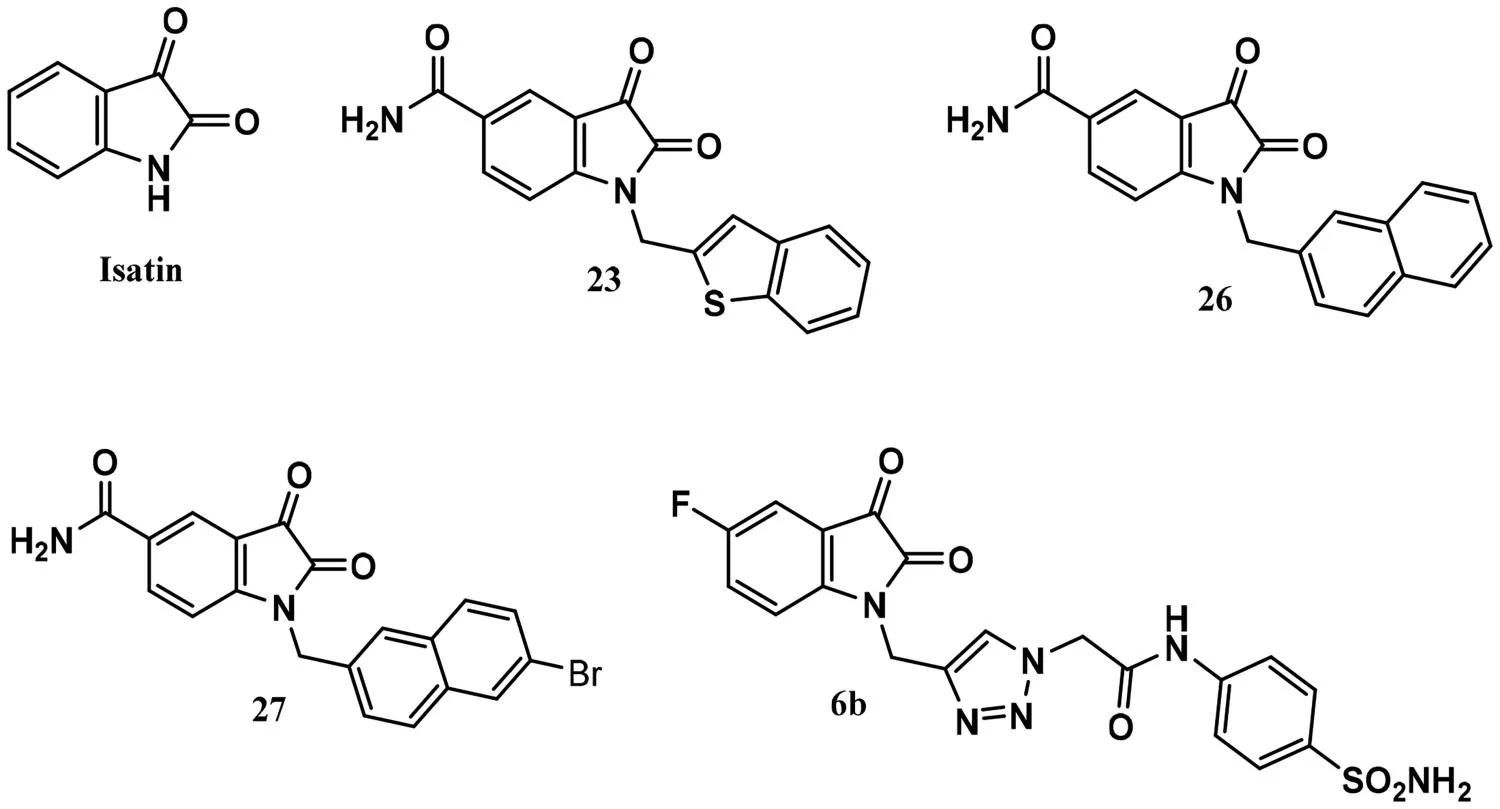
Structures of isatin and some reported main SARS-CoV-2 3CL^pro^ inhibitors.

Isatin (1H-indole-2, 3-dione) is an opulent heterocycle that has been proven to provide tremendous opportunities in the area of drug discovery. Over the last fifty years, suitably functionalised isatin has shown remarkable and broad-spectrum antiviral properties^9^. For example, the antiviral activities for diverse isatin derivatives were reported against HIV^10–12^, arbovirus^13^, chikungunya virus^14^, herpes simplex virus^15^, coxsackievirus^16^, poxvirus^17^, and influenza virus^18^. Furthermore, many investigations have been conducted in order to afford isatin analogues as effective inhibitors of SARS-CoV main protease^19–22^. Recently, Liu et al assessed the antiviral activity of other new N-substituted isatin-based molecules, by targeting SARS-CoV-2 3CL^pro^. The three most potent compounds^23–25^ inhibited SARS-CoV-2 3CL^pro^ with IC_50_’s of 53 nM, 45 nM and 47 nM, respectively. They found that isatin compounds with a carboxamide substitution at the C-5 position and aromatic substitution at N-1 position were strong inhibitors of SARS-CoV-2 3CL^pro^^23^. Regrettably, their cellular antiviral activities were not further assessed. El Naggar et al also developed novel triazolo-isatins for inhibiting main protease of the virus, a crucial enzyme for its replication in the host cells. Particularly, sulphonamide **6b** showed promising inhibitory activity with an IC_50_ of 0.249 µM. Significantly, **6b** inhibited viral cell proliferation with an IC_50_ of 4.33 µg/ml, and was non-toxic to VERO-E6 cells (CC_50_ of 564.74 µg/ml) displaying a selectivity index of 130.4. In silico analysis of **6b** disclosed its ability to interact with key residues in the enzyme active site, supporting the obtained *in vitro* findings^26^. These SARS-CoV-2 3CL^pro^ inhibitors are shown in Figure 1.

**Figure 2.**
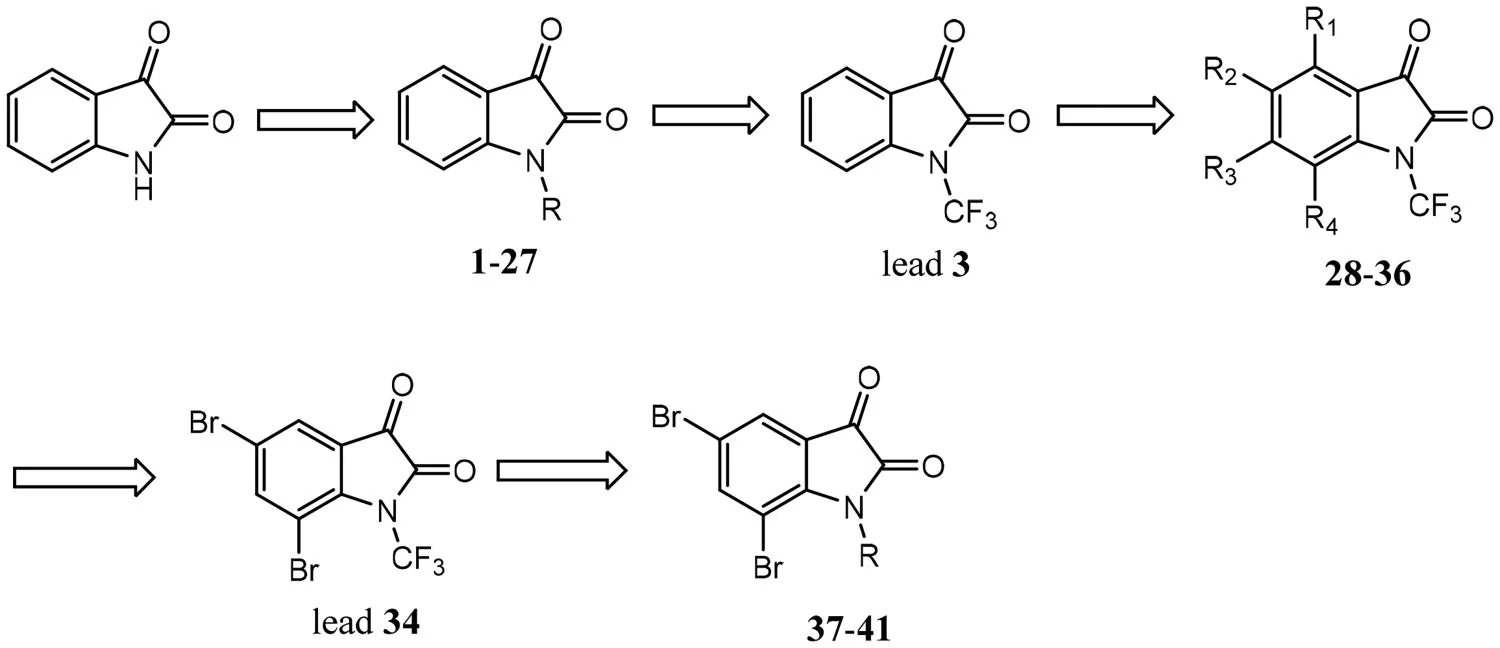
The design ideas of novel isatin derivatives.

**Figure 3.**
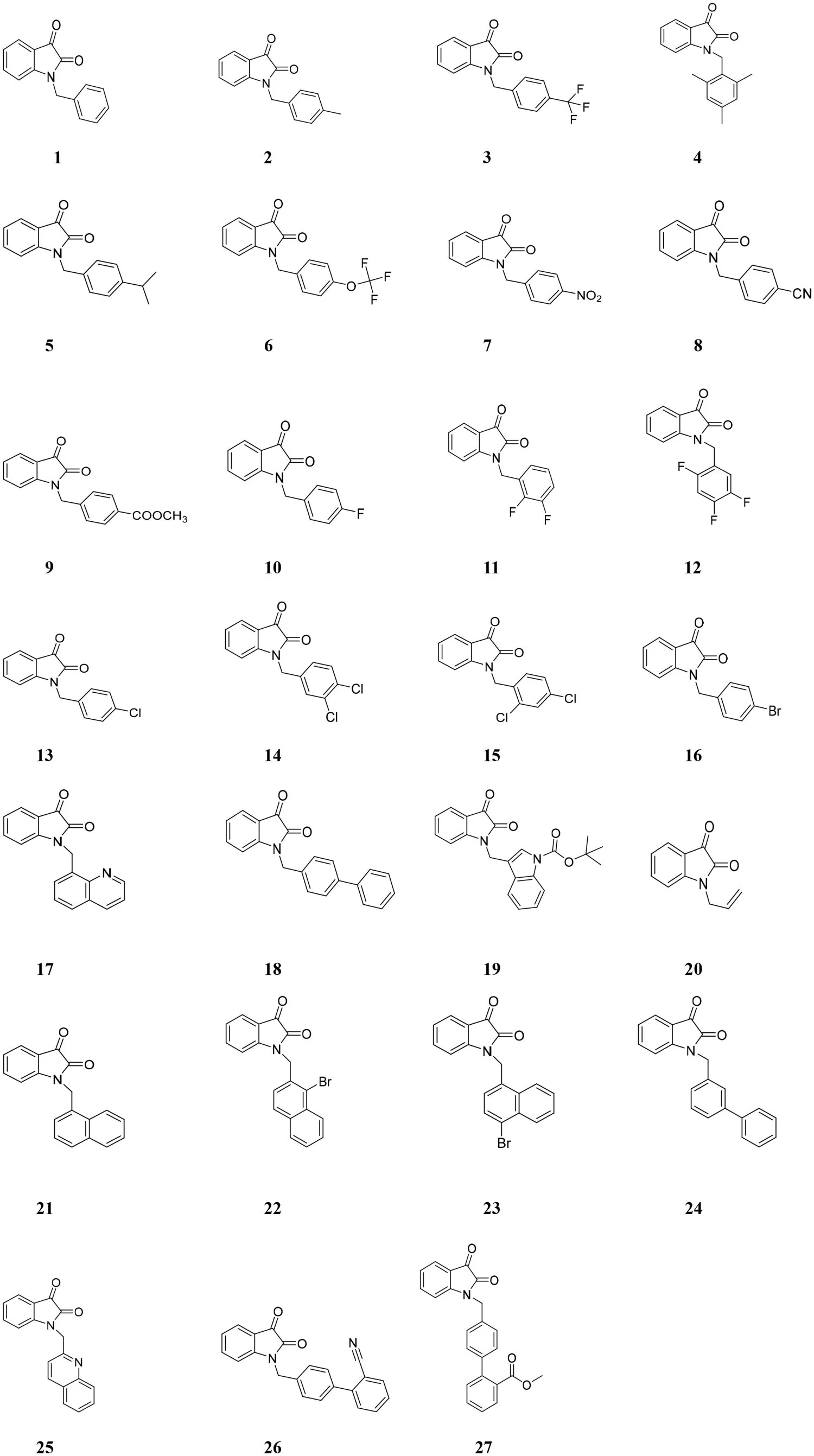
The structures of compounds **1–27**.

**Figure 4.**
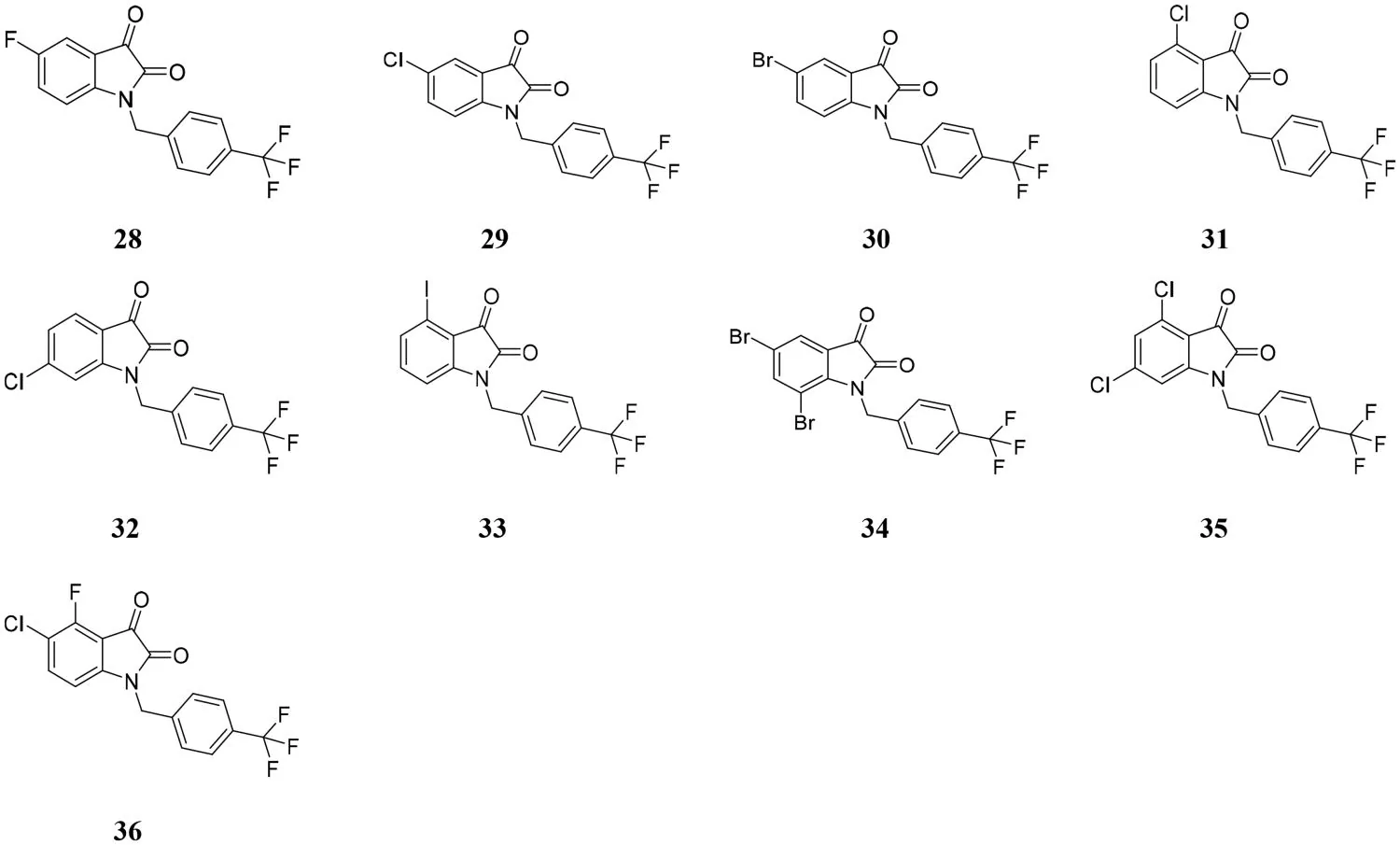
The structures of compounds **28–36**.

**Figure 5.**
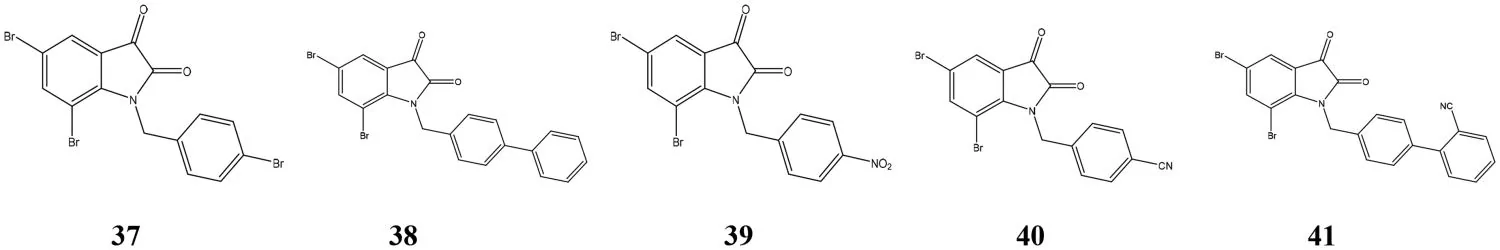
The structures of compounds **37–41**.

In order to verify whether isatin derivatives could inhibit SARS-CoV-2 3CL^pro^ and find novel isatin derivatives, we decided to synthesise a series of novel isatin derivatives by incorporating phenyl, biphenyl, naphthyl, quinolyl and indolyl moieties at the N-1 position of isatin or halogen at the isatin ring (Figure. 2), and tested their inhibitory effects against SARS-CoV-2 3CL^pro^. Their chemical structures were shown in Figures. 3–5, respectively.

## Results and discussion

### Chemistry

**Scheme 1.**
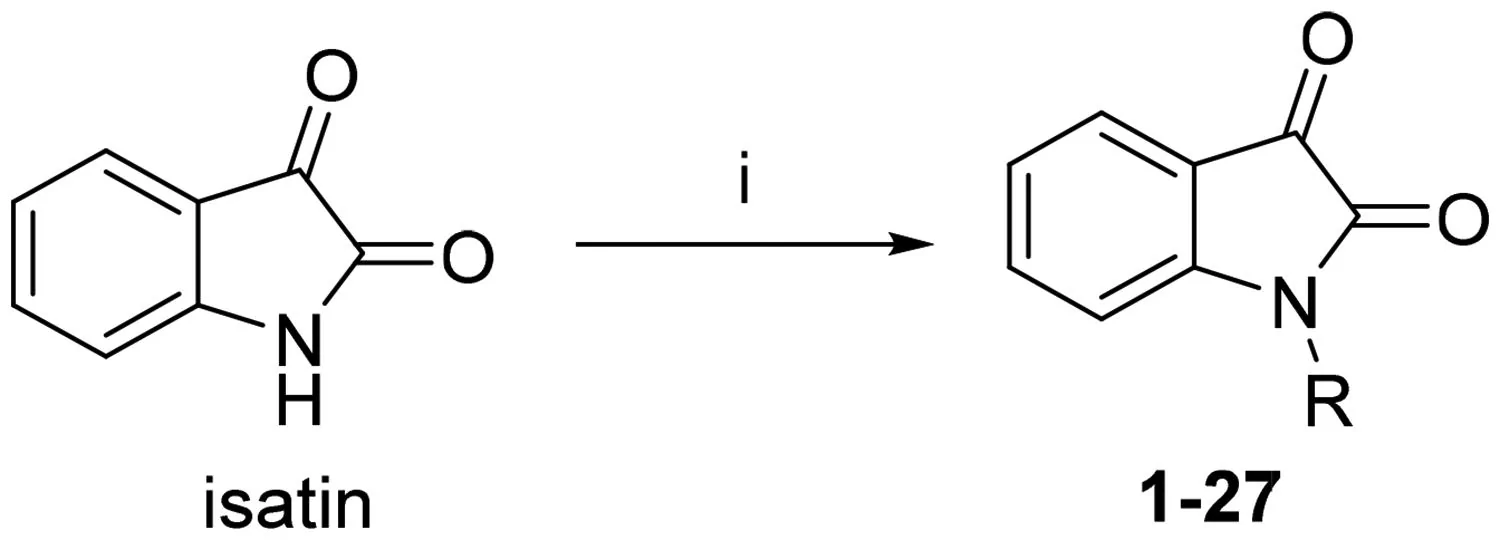
Reagents and conditions: (i) RBr, NaOH, DMF, room temperature, 0.5 h, 85% - 93%.

**Scheme 2.**
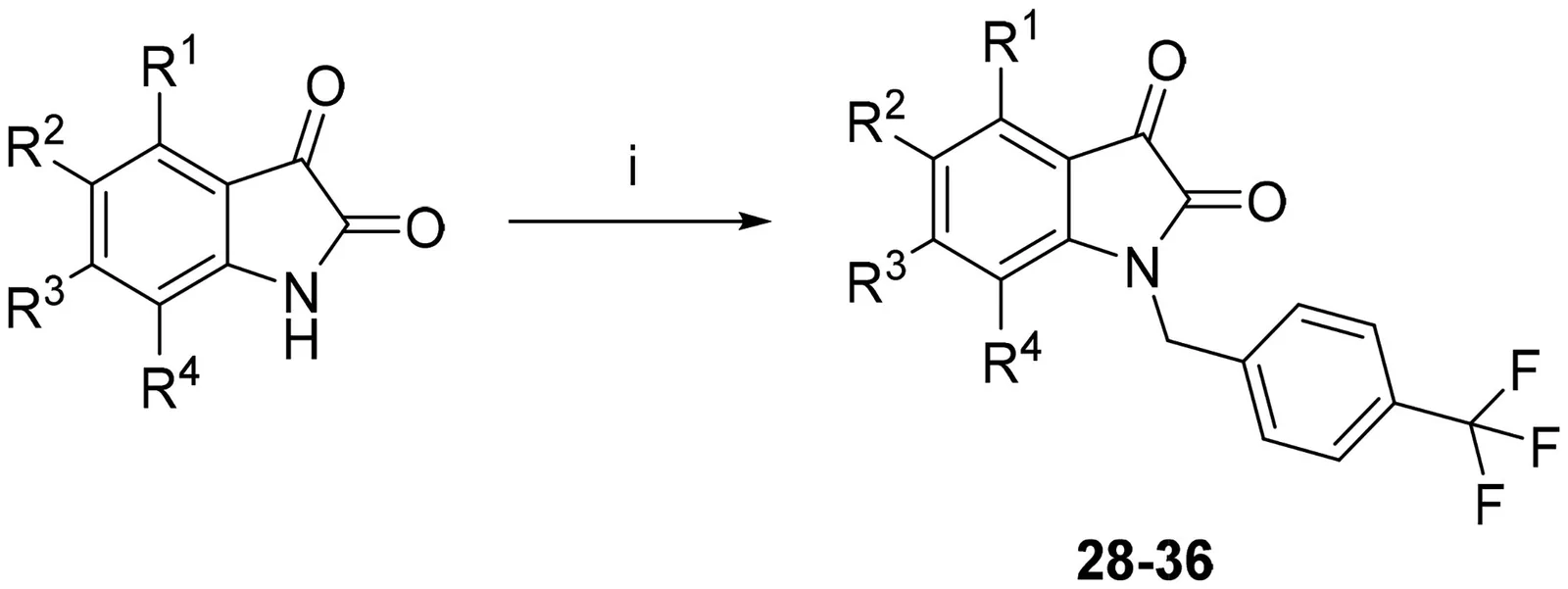
Reagents and conditions: (i)1-(bromomethyl)-4-(trifluoromethyl) benzene, NaOH, DMF, room temperature, 0.5 h, 85% - 91%.

**Scheme 3.**
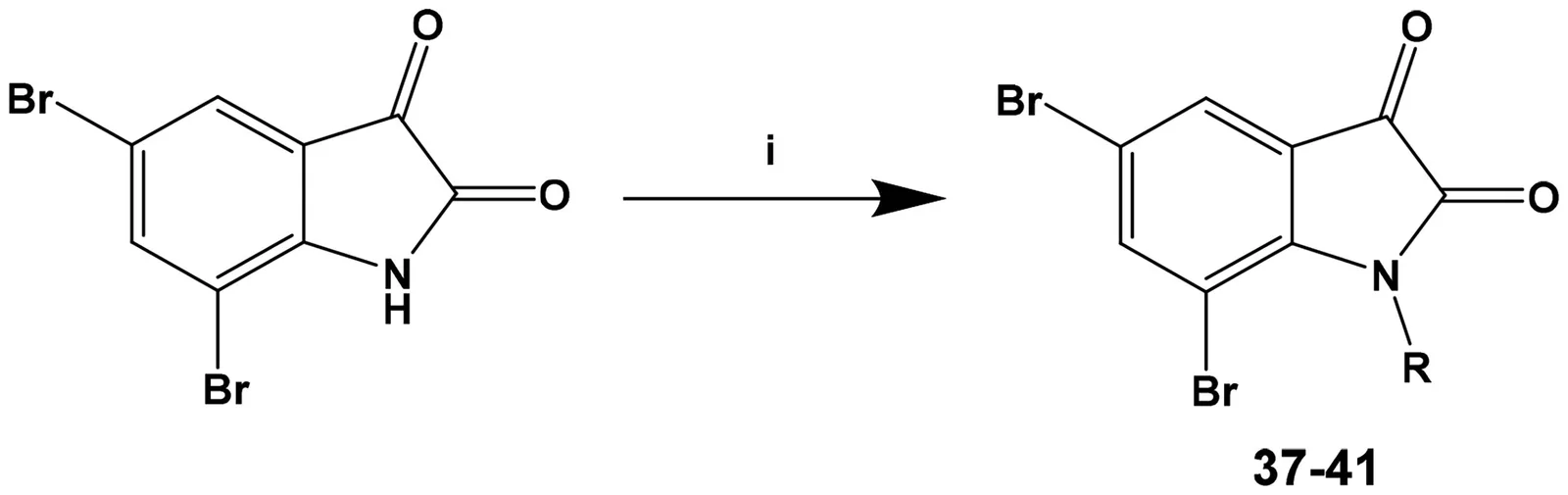
Reagents and conditions: (i) RBr, NaOH, DMF, room temperature, 0.5 h, 86% - 90%.

The synthesis of compounds **1–41** were shown in Schemes 1–3, respectively. Novel isatin derivatives were synthesised by treating isatin or halogen substituted isatin derivatives with brominated derivatives or 1-(Bromomethyl)-4-(trifluoromethyl) benzene in a suitable solvent to form adduct in high yields (more than 85%), respectively. The analytical and spectral data of these title compounds agreed with the desired chemical structures.

### SARS-CoV-2 3CL^pro^ inhibitory assay

In vitro inhibition activity assay was performed essentially as described by the manufacturer (Beyotime, catalogue # P0312M)^27^. Ebselen was used as a positive control for the enzyme assay. The compounds with 50% or more inhibition were considered active. Inhibition of SARS-CoV-2 3CL^pro^ by these active compounds at various concentrations were further measured, and their IC_50_ (the concentration that causes 50% of SARS-CoV-2 3CL^pro^ inhibition) values were determined. From Table 1, as a parent compound, isatin was inactive. However, introduction of phenyl, biphenyl, naphthyl, quinolyl and indolyl moieties into N-1 position of isatin led to an increase in inhibition activity against SARS-CoV-2 3CL^pro^, respectively. Among 27 compounds, 8 compounds showed inhibition activity over 50% at 50 µM, such as compounds **3**, **7**, **13**, **14**, **16**, **23, 26** and **27**. Interestingly, only compound **3**(R = 4-trifluoromethylbenzyl, IC_50_=6.758 ± 1.162 µM) showed higher activity against SARS-CoV-2 3CL^pro^ than other compounds, while compound **7** (R = 4-nitrobenzyl, IC_50_=18.307 ± 3.191 µM) only had moderate inhibition activity. The results showed that 4-trifluoromethylphenyl group at N-1 position is essential for the high inhibition activity, and superior to other hydrophobic aromatic substituents like biphenyl, naphthyl, quinolyl and indolyl groups. Additionally, trifluoromethyl group were also superior to other substituents at the 4-position of benzyl group, such as halogen, carbomethoxy group, methyl group, isopropyl group, trifluoromethoxy group, cyano group, nitro group and so on.

**Table 1.** Inhibition activities of compounds **1–27** against SARS-CoV-2 3CL^pro^.

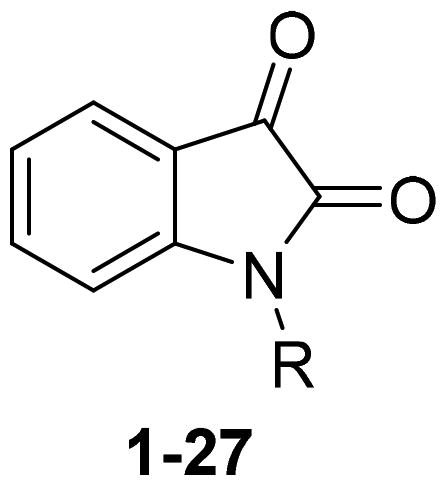		
Compound	R	^a^IC_50_(µM) or percentage (%) of inhibition at 50 µM
Ebselen		0.354 ± 0.030
**1**	benzyl	4.16 at 50 µM
**2**	4-methylbenzyl	28.79 at 50 µM
**3**	4-trifluoromethylbenzyl	6.758 ± 1.162
**4**	2,4,6-trimethylbenzyl	37.10 at 50 µM
**5**	4-isopropylbenzyl	49.69 at 50 µM
**6**	4-trifluoromethoxybenzyl	45.60 at 50 µM
**7**	4-nitrobenzyl	18.307 ± 3.191
**8**	4-cyanobenzyl	4.47 at 50 µM
**9**	4-carbomethoxybenzyl	24.76 at 50 µM
**10**	4-fluorobenzyl	36.50 at 50 µM
**11**	2,3-difluorobenzyl	45.07at 50 µM
**12**	2,4,5-trifluorobenzyl	48.38 at 50 µM
**13**	4-chlorobenzyl	52.98 at 50 µM
**14**	3,4-dichlorobenzyl	52.67 at 50 µM
**15**	2,4-dichlorobenzyl	33.54 at 50 µM
**16**	4-bromobenzyl	66.03 at 50 µM
**17**	8-quinolylmethyl	0.72 at 50 µM
**18**	4-phenylbenzyl	13.95 at 50 µM
**19**	1-carboxylic acid tert-butyl ester-indole-3-methyl	10.07 at 50 µM
**20**	allyl	9.93 at 50 µM
**21**	1-naphthylmethyl	26.34 at 50 µM
**22**	1-bromo-2-naphthylmethyl	47.07 at 50 µM
**23**	1-bromo-4-naphthylmethyl	68.24 at 50 µM
**24**	3-phenylbenzyl	35.40 at 50 µM
**25**	2-quinolylmethyl	43.64 at 50 µM
**26**	4-(2-cyanophenyl)-benzyl	73.30 at 50 µM
**27**	4-(2-carbomethoxyphenyl)-benzyl	66.31 at 50 µM

^a^IC_50_: the concentration that causes 50% of SARS-CoV-2 3CL^pro^ inhibition.

The SARs of isatin derivatives as SARS-CoV-2 3CL^pro^ inhibitors revealed that electron withdrawing groups are favourable at C-5 of isatin ring, such as I, Br, F, CONH_2_ and SO_2_NHR^9^. Therefore, compound **3** was chose as a lead compound for further study. Then, compounds **28–36** bearing halogen at the isatin ring were prepared and evaluated for their inhibitory effects against SARS-CoV-2 3CL^pro^ (Scheme 2 and Table 2).

**Table 2.** Inhibition activities of compounds **28–36** against SARS-CoV-2 3CL^pro^.

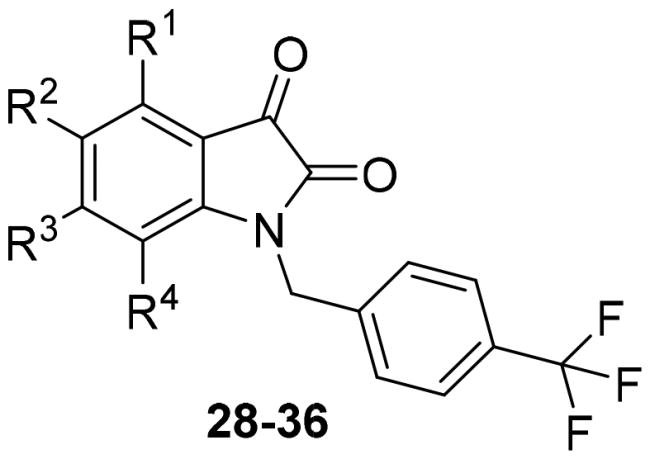					
Compound	R^1^	R^2^	R^3^	R^4^	^a^IC_50_(µM) or percentage (%) of inhibition at 50 µM
Ebselen					0.354 ± 0.030
**28**	H	F	H	H	10.479 ± 0.773
**29**	H	Cl	H	H	1.212 ± 0.254
**30**	H	Br	H	H	1.399 ± 0.228
**31**	Cl	H	H	H	94.46 at 50 µM
**32**	H	H	Cl	H	12.408 ± 2.873
**33**	I	H	H	H	89.51 at 50 µM
**34**	H	Br	H	Br	0.363 ± 0.045
**35**	Cl	H	Cl	H	3.397 ± 2.820
**36**	F	Cl	H	H	58.62 at 50 µM

^a^IC_50_: the concentration that causes 50% of SARS-CoV-2 3CL^pro^ inhibition.

**Figure 6.**
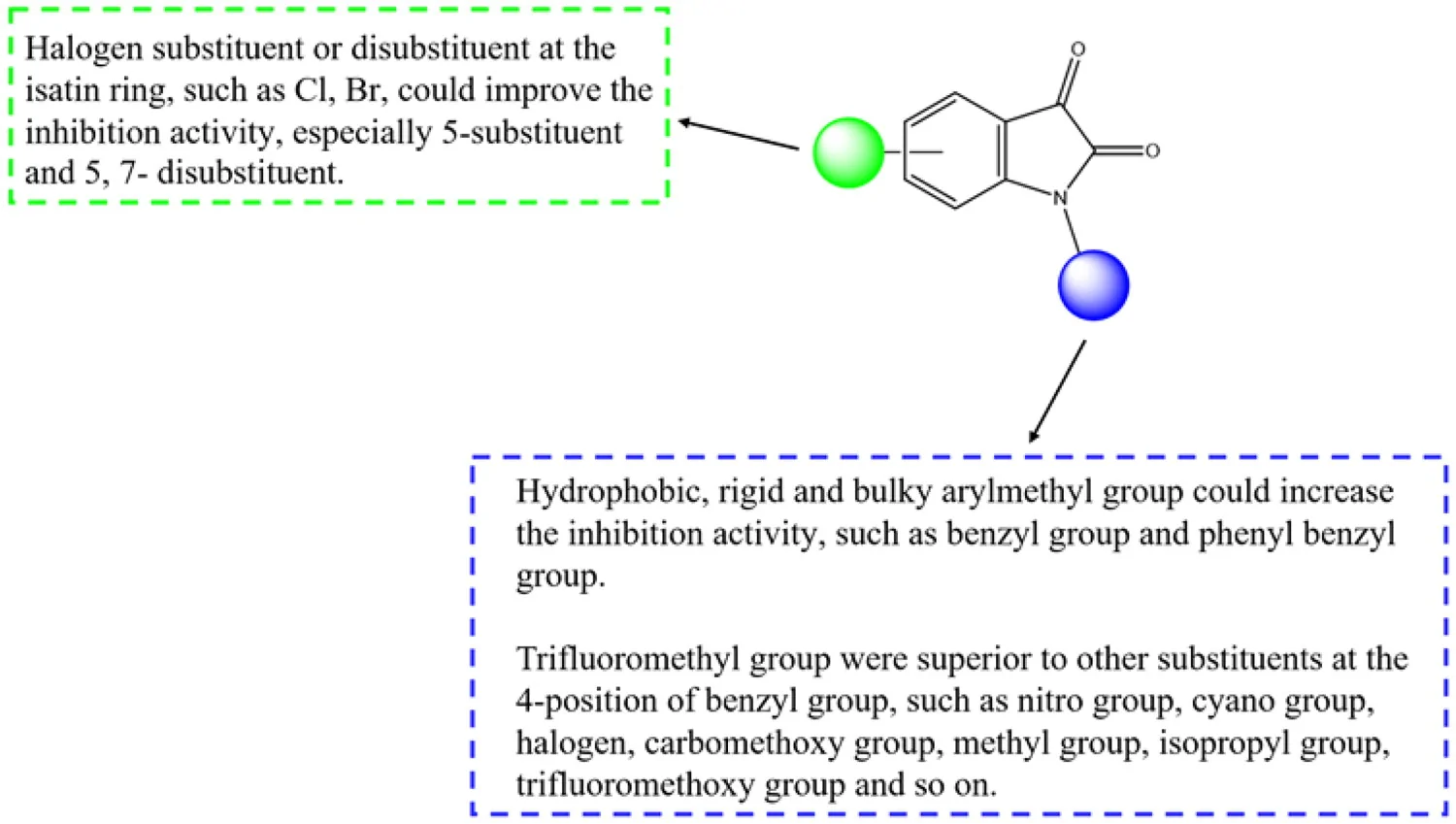
The SARs of novel isatin derivatives as SARS-CoV-2 3CL^pro^ inhibitors.

From Table 2, compounds **31**, **33** and **36** showed inhibition activity over 50% at 50 µM, but their IC_50_ values were not determined. Compound **32** (R^3^=Cl, IC_50_=12.408 ± 2.873 µM) had moderate activities against SARS-CoV-2 3CL^pro^, while compound **35** (R^1^=Cl and R^3^=Cl, IC_50_=3.397 ± 2.820 µM) showed higher activities against SARS-CoV-2 3CL^pro^ in comparison with compound **3.** As for compounds **28–30** bearing halogen on the C-5 position of isatin ring, compound **29** (R^2^=Cl, IC_50_=1.212 ± 0.254 µM) and compound **30** (R^2^=Br, IC_50_=1.399 ± 0.228 µM) exhibited stronger activities against SARS-CoV-2 3CL^pro^ than compound **3**, while compound **28** (R^2^=F, IC_50_=10.479 ± 0.773 µM) only had moderate activities against SARS-CoV-2 3CL^pro^. Interestingly, compound **34** (R^2^=Br and R^4^=Br) demonstrated an IC_50_ of 0.363 ± 0.045 µM against SARS-CoV-2 3CL^pro^, which is found to be equipotent to the positive drug Ebselen (IC_50_=0.354 ± 0.030 µM). These results indicated that introduction of halogen at the isatin ring could improve the inhibition activity of SARS-CoV-2 3CL^pro^, which supported the previous study results^9^^,^^23^^,^^26^. Moreover, introduction of electron withdrawing groups at the C-5 and C-7 of isatin ring, such as bromo group, might be more favourable for the inhibition activity. On the basis of the above results, compounds **37–41** bearing bromo groups at the C-5 and C-7 of isatin ring were prepared and evaluated for their inhibitory effects against SARS-CoV-2 3CL^pro^ (Scheme 3 and Table 3). As we expected, compounds **37–41** effectively inhibited SARS-CoV-2 3CL^pro^ with IC_50_ values spanning from 0.459 ± 0.019 to 4.844 ± 0.408 µM. Notably, compound **39** also displayed stronger inhibitory activity (IC_50_= 0.459 ± 0.019 µM), next to compound **34** and Ebselen. To this end, the structure-activity relationships (SARs) is presented in Figure 6 for further rational development of isatin derivatives with higher potency against SARS-CoV-2 3CL^pro^.

**Table 3.** Inhibition activities of compounds **37–41** against SARS-CoV-2 3CL^pro^.

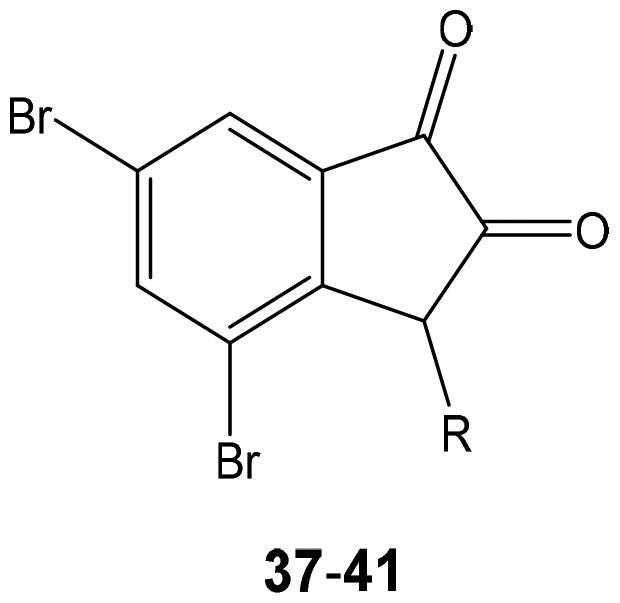		
Compound	R	^a^IC_50_ (µM)
Ebselen		0.354 ± 0.030
**37**	4-bromobenzyl	1.287 ± 0.127
**38**	4-phenylbenzyl	—
**39**	4-nitrobenzyl	0.459 ± 0.019
**40**	4-cyanobenzyl	4.844 ± 0.408
**41**	4-(2-cyanophenyl)-benzyl	1.606 ± 0.044

^a^IC_50_: the concentration that causes 50% of SARS-CoV-2 3CL^pro^ inhibition. Data are expressed as mean ± SD from triplicate determination from three independent experiments.—: no calculation.

### The SARS-CoV-2 inhibitory assay (cell-based)

Since some compounds showed the strong inhibitory effects against SARS-CoV-2 3CL^pro^, their cellular antiviral activities were further assessed^24^. As shown in Table 4, the compounds **34** (EC_50_=0.402 ± 0.021 *μ*M), **35** (EC_50_=2.218 ± 0.250 *μ*M), **37** (EC_50_=0.795 ± 0.154 *μ*M), **39** (EC_50_=0.443 ± 0.061 *μ*M), **40** (EC_50_= 3.607 ± 0.058 *μ*M), and **41** (EC_50_=0.847 ± 0.085 *μ*M) have comparable antiviral activity to Ebselen (EC_50_=0.450 ± 0.075 *μ*M), while compounds **29** (EC_50_= 24.463 ± 6.519 *μ*M) and **30** (EC_50_=10.845 ± 1.108 *μ*M) demonstrated moderate activity against SARS-CoV-2 in Vero-E6 cells. The results also further verified that introduction of electron withdrawing groups at the C-5 and C-7 of isatin ring, such as bromo group, might be more favourable for the inhibition activity.

**Table 4.** Inhibition activities of the designed active compounds against SARS-CoV-2.

Compound	^a^EC_50_ (µM)	^b^CC_50_(µM)	^c^SI(CC_50_/ EC_50_)
Ebselen	0.450 ± 0.075	69.651 ± 8.892	155
**29**	24.463 ± 6.519	285.546 ± 16.127	12
**30**	10.845 ± 1.108	163.652 ± 14.895	15.
**34**	0.402 ± 0.021	68.470 ± 6.162	170
**35**	2.218 ± 0.250	168.386 ± 15.895	76
**37**	0.795 ± 0.154	35.951 ± 1.703	45
**39**	0.443 ± 0.061	48.075 ± 2.741	108
**40**	3.607 ± 0.058	82.952 ± 4.961	23
**41**	0.847 ± 0.085	30.263 ± 1.345	33

^a^EC_50_: the novel compound concentration that causes 50% of SARS-CoV-2 virus (strain IVCAS 6.7512) inhibition.

^b^CC_50_: novel compound concentration required to cause 50% death of uninfected VeroE6 cells. ^c^SI: selectivity index as CC_50_/EC_50_.

Remarkably, compound **34** exerted promising cell growth inhibitory activity with IC_50_=0.402 ± 0.021 *μ*M (SI = 170). This outcome is most likely associated with 3**4**’s capacity to efficiently inhibit the SARS-CoV-2 3CL^pro^, as described above.

### Drug likeness

Next, we analysed the druglike nature of the designed active compounds to support drug discovery. Also, we presented the bioavailability score for all compounds obtained via SwissADME web tool^25^. The corresponding results are listed in Table 5. All compounds have showed ADME parameters in an acceptable range and possess significant drug-like characteristics based on Lipinski’s rule of 5^28^. The findings indicate that these active compounds might be very good candidates as potential inhibitor for SARS-CoV-2 3CL^pro^, but the results still need to be verified via further in *vitro* or *vivo* ADME analysis in the future studies.

**Table 5.** In-silico predicted ADME properties of the designed active compounds.

compounds	Molecular weight (≤ 500 g/mol)	Log P (≤ 5)	H-Bond donor (≤ 5)	H-bond acceptor (≤ 10)	Bioavailability Score	Log S (ESOL)	Solubility (mg/ml)	Lipinski Rule
**29**	339.70	3.76	0	5	0.55	−4.60	8.58 **×** 10^-3^	Yes; 0 violation
**30**	384.15	3.86	0	5	0.55	−4.91	4.72 **×** 10^-3^	Yes; 0 violation
**34**	463.04	4.45	0	5	0.55	−5.82	7.03 **×** 10^-4^	Yes; 0 violation
**35**	374.14	4.26	0	5	0.55	−5.19	2.44 **×** 10^-3^	Yes; 0 violation
**37**	473.94	4.04	0	2	0.55	−5.89	6.17 **×** 10^-4^	Yes; 0 violation
**39**	440.04	2.81	0	4	0.55	−5.02	4.16 **×** 10^-3^	Yes; 0 violation
**40**	420.05	3.17	0	3	0.55	−4.91	5.11 **×** 10^-3^	Yes; 0 violation
**41**	496.15	4.49	0	3	0.55	−6.42	1.89 **×** 10^-4^	Yes; 0 violation

### Reversibility of inhibition

**Figure 7.**
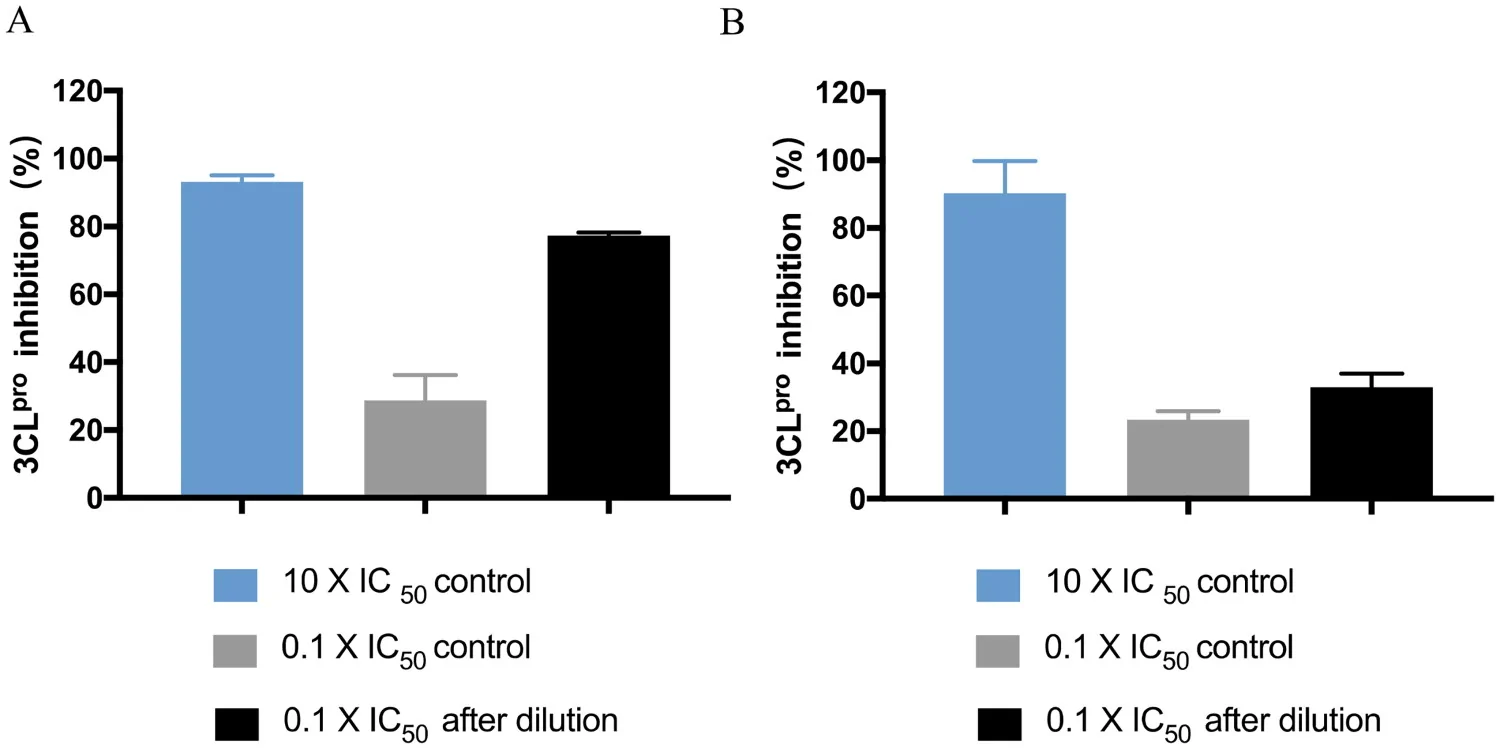
Assessment of the reversible mechanism of inhibition with compound **34**. SARS-CoV-2 3CL^pro^ was incubated with compound **34** in a jump dilution assay. (A) The enzymatic inhibitions of compound **34** just 10 min after the compound dilution with substrate(*n* = 3). (B) The enzymatic inhibitions of compound **34** just 2 h after the compound dilution with substrate (*n* = 3). Enzymatic inhibitions (%) are calculated as mean ± SD of triplicate. Data are representative of three other experiments.

To elucidate the binding mode of compound **3**4 with the SARS-CoV-2 3CL^pro^, a jump dilution assay^29^ is used to evaluate the reversible of inhibition. To that aim, the compound **34** was pre-incubated at 10 times its IC_50_ with SARS-CoV-2 3CL^pro^. Then, incubates were quickly diluted to 1/100 with substrate solution before measuring the fluorescence kinetics. Inhibition was compared to control standard incubations at 10 × IC_50_ and 0.1 × IC_50_ final concentrations of compound **34**. As shown in Figure 7, during the first 10 min after jump dilution, the enzyme was inhibited by approximately 80% (Figure 7A), a value similar to the 10 × IC_50_ control value. After 2 h, enzyme activity was progressively recovered, with net inhibition of only approximately 30% (Figure. 7B).

### Surface plasmon resonance (SPR) technique

**Figure 8.**
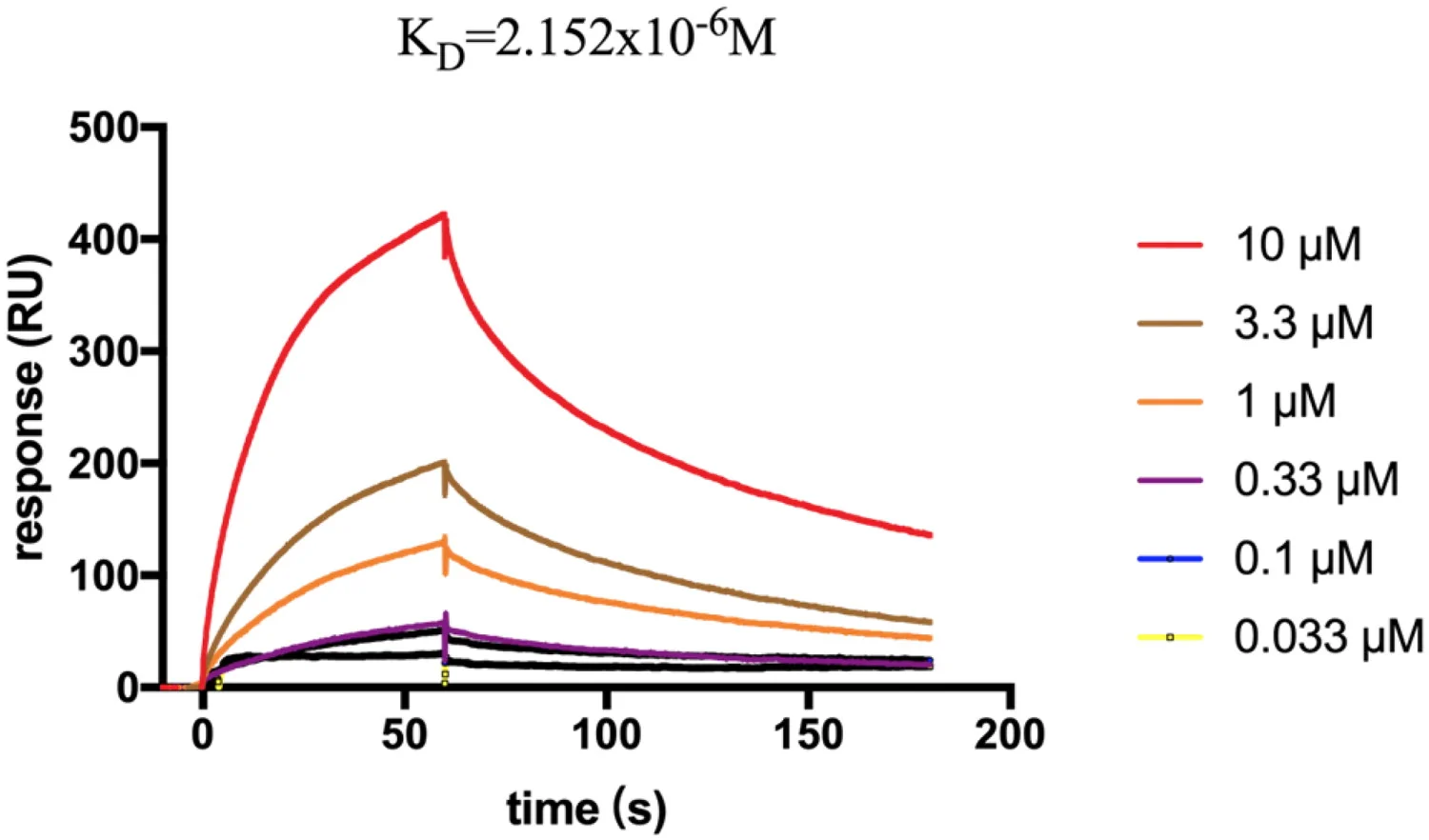
Surface plasmon resonance sensor grams of compound **34** for its interactions with SARS-CoV-2 3CL^pro^.

The binding of compound **34** to SARS-CoV-2 3CL^pro^ was further studied using SPR spectroscopy, which allowed measurement of their interactions in real time^30^. Here, the interactions were examined for compound **34** that showed high SARS-CoV-2 3CL^pro^ inhibitory activity in the initial *in-vitro* screening. Compound **34** bound to the SARS-CoV-2 3CL^pro^ immobilised on the chip (Figure 8). The two-state kinetics model provided a fit for the compound **34**–SARS-CoV-2 3CL^pro^ interaction, which indicated a *K*_D_ of 2.152 × 1 0 –6 M. The sensor grams suggested that that the dissociation of compound **34** from SARS-CoV-2 3CL^pro^ appeared to occur in two steps, as an initial rapid decrease in the SPR response that was followed by a slow dissociation. This is likely to be the consequence of multiple binding sites on SARS-CoV-2 3CL^pro^ with different affinities. The observed K_D_ (2.152 × 1 0 –6 M) exceeds the IC_50_ (0.363 μM), indicating that binding affinity underestimates functional potency. This discrepancy is consistent with a two-step inhibition mechanism where initial inhibitor encounter (K_D_) is followed by a slow conformational transition to a high-affinity complex that dominates the functional assay. Alternatively, slow dissociation kinetics (k off **≪** k on) may result in accumulation of the inhibited state during the extended incubation periods used for activity measurements. The data suggest that compound **34** acts as a time-dependent or tight-binding inhibitor rather than following simple rapid-equilibrium competitive inhibition. IC_50_ is usually measured after the reaction reaches a steady state, reflecting the inhibitory effect of the final stable complex. If K_D_ is determined by techniques such as Surface Plasmon Resonance (SPR) or Isothermal Titration Calorimetry (ITC), it may only capture the dissociation constant of the first rapid binding step, without reflecting the stronger binding affinity brought by the second conformational change. In this case, K_D_ (the dissociation constant of the first step) will be larger than the true overall binding constant, while IC_50_ is closer to the overall inhibitory effect, resulting in the phenomenon of K_D_>IC_50_.

### Molecular docking analysis

**Figure 9.**
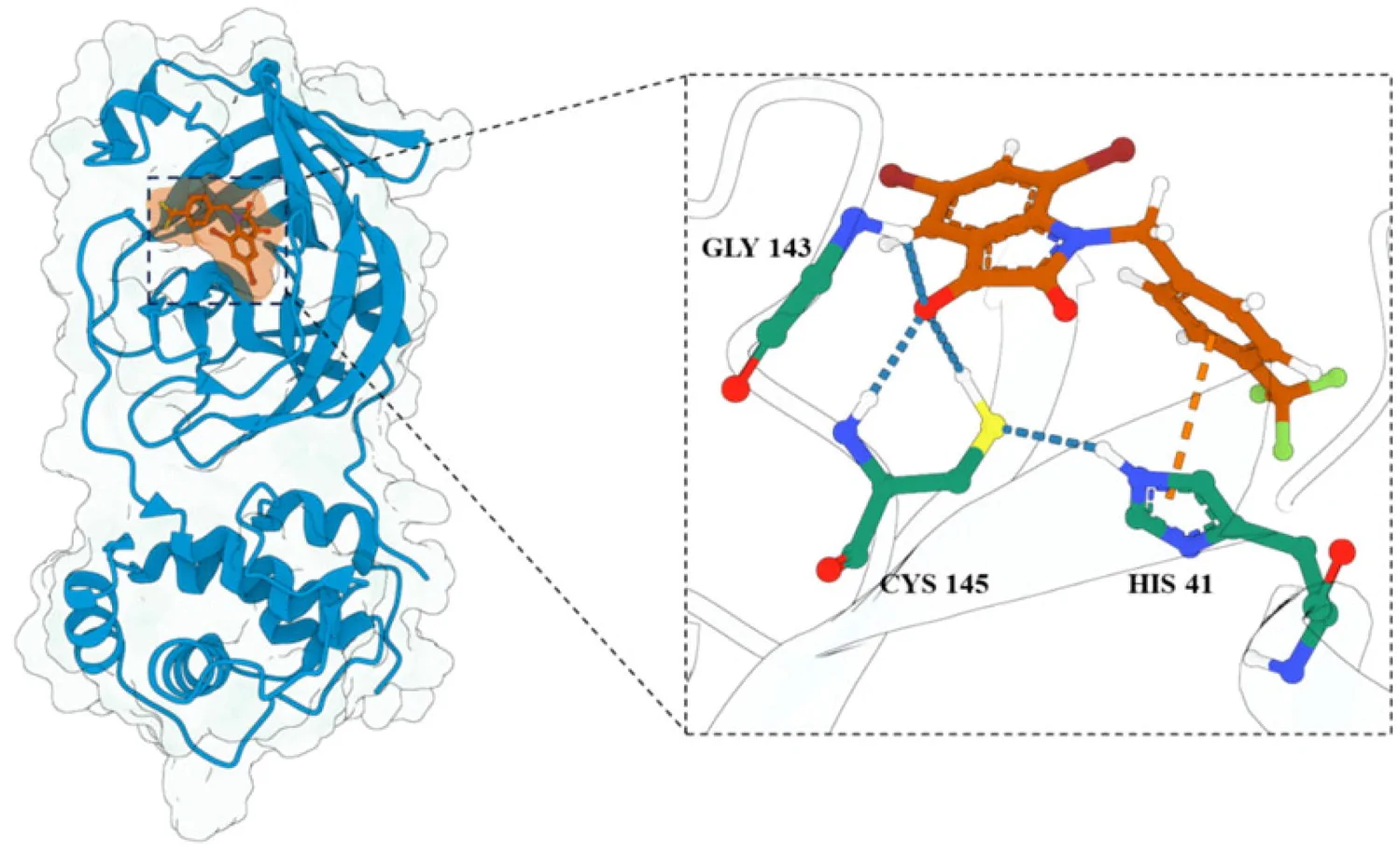
Compound **34** (brown stick) docked into the active pocket of SARS-CoV-2 3CL^pro^ (PDB ID 6LU7); The hydrogen bonds and the π-π stacking interactions between compound **34** and SARS-CoV-2 3CL ^pro^ are shown in blue dashed lines and brown dashed lines, respectively.

Furthermore, the crystal structure of SARS-CoV-2 3CL^pro^ (PDB ID 6LU7)^5^ was used for molecular docking. As shown in Figure 9, compound **34** forms multiple interactions with SARS-CoV-2 3CL^pro^. Firstly, compound **34** exhibits excellent shape complementarity with the protein’s active site. Additionally, the carbonyl oxygen atom in its core scaffold forms dynamic hydrogen bonds with Gly143 and Cys145 of the main protease. The benzyl substituent on the side chain engages in π-π stacking interactions with the HIS41 side chain, further enhancing the overall stability of the complex. Overall, this binding mode demonstrates strong binding stability and specificity. It is also reported that the carbonyl oxygen atom in its core scaffold forms dynamic hydrogen bonds with Cys145 of the main protease^23^^,^^26^, supporting our docking results. However, the dynamic stability of the binding conformation still requires further validation through molecular dynamics simulations.

### Molecular dynamics (MD)

After 100 ns of molecular dynamics simulation, we conducted an in-depth analysis of the simulation trajectory between compound **34** and SARS-CoV-2 3CL^pro^ (PDB ID 6LU7)^31^. First, we extracted the Root Mean Square Deviation (RMSD) data from the simulation. RMSD is a key indicator for evaluating the conformational stability of proteins and ligands in molecular dynamics simulations, quantifying the deviation of the structure from a reference conformation during the simulation. The lower the RMSD value, the more stable the conformation is relative to the reference.

**Figure 10.**
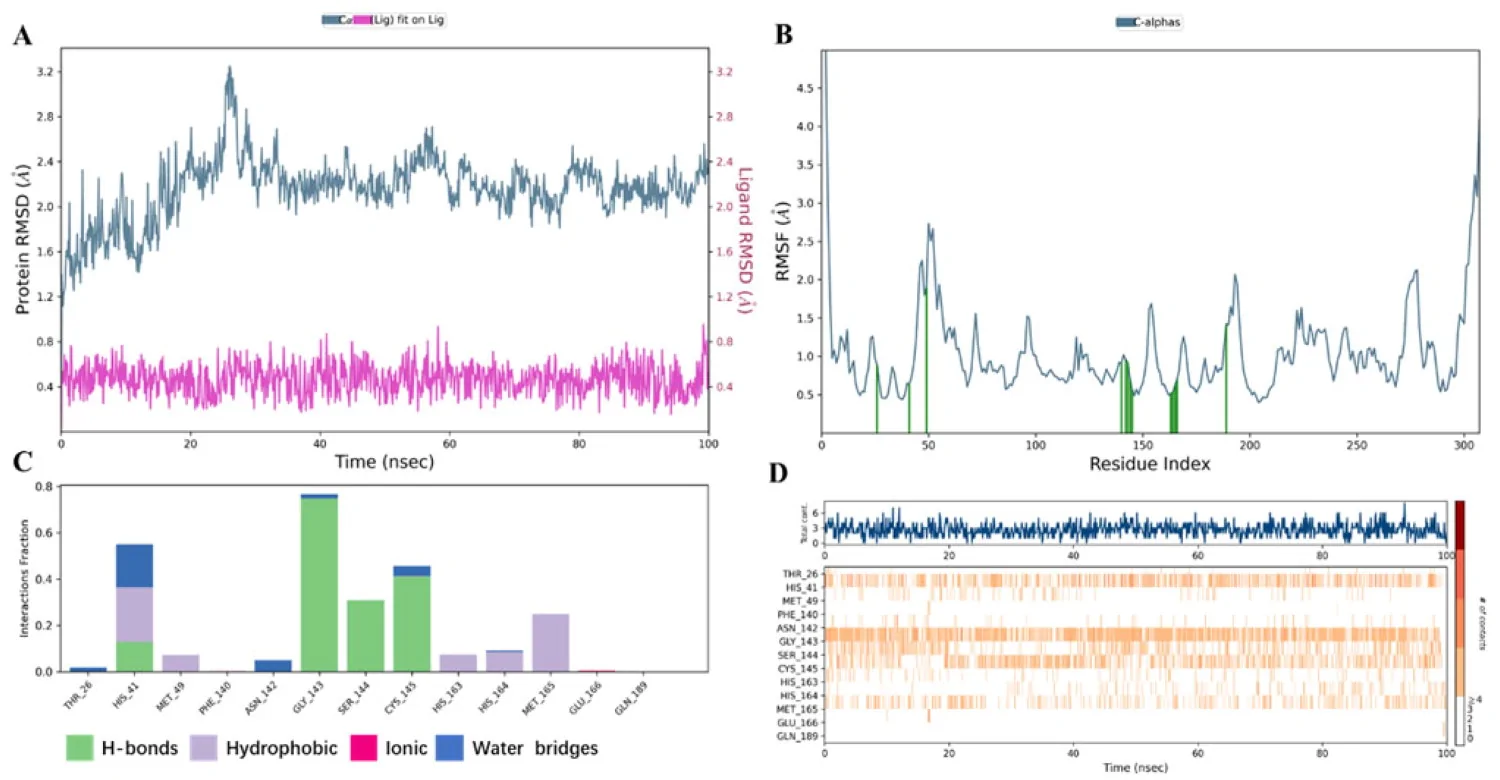
The 100 ns MD simulation of compound **34**. (A) The RMSD plot of SARS-CoV-2 3CL^pro^ (6LU7)-compound **34** complex during the 100 ns simulations. (B) The RMSF diagram of SARS-CoV-2 3CL^pro^-compound **34** complex at the end of the 100 ns simulations (protein residues that interact with the ligand are marked with green-colored vertical bars.). (C) The bar diagrams of SARS-CoV-2 3CL^pro^ -compound **34** complex contacts during 100 ns simulations (green represents hydrogen bonds, blue represents water bridges, purple represents hydrophobic contacts, and rose-red represents ionic interactions). (D) Non-covalent interactions between **34** and protein residues over time and frequency during the 100 ns simulation.

As shown in Figure. 10A, the RMSD of the protein rapidly increased in the early stage of the simulation and then gradually levelled off. In the subsequent approximately 70 ns of simulation time, it remained within the range of 2.0–2.4 Å without significant structural rearrangement. This indicated that the protein backbone maintained a high degree of structural stability overall, without undergoing denaturation or drastic conformational changes. It was worth noting that the RMSD value of the ligand remained within a low range (0.3–0.8 Å) throughout the entire 100 ns simulation, with no obvious upward fluctuation trend. This suggested that compound **34** was stably located at the binding site and did not detach from the binding pocket or undergo drastic conformational changes. The RMSD analysis results showed that both the protein and compound **34** exhibited good stability during the molecular dynamics simulation, with a stable and persistent binding state, indicating that the complex system had a stable binding mode.

Meanwhile, we extracted the RMSF (Root Mean Square Fluctuation) of the protein and compound **34**. RMSF analysis is used in molecular dynamics simulations to evaluate the fluctuation of protein residues and ligand atoms. As can be seen from Figure. 10B, the RMSF values of most protein residues were below 1.5 Å, with higher fluctuations only observed at the protein termini. This indicated that the majority of the protein backbone maintained good conformational stability throughout the simulation. The residues that directly contacted compound **34** (binding site residues) were highlighted in green in the figure. These residues generally showed fluctuations below 1.0 Å in the RMSF curve, indicating that compound **34** formed stable interactions with the protein, without causing significant perturbation in the binding site region. The binding site remained highly stable during the molecular dynamics process. This result further corroborated the conclusions drawn from the aforementioned RMSD analysis.

To identify the key residues involved in the binding process between the protein and compound **34**, we analysed the interaction frequency in the molecular dynamics simulation trajectories. Figure. 10C showed the frequency interactions fraction with which each key residue formed different types of non-covalent interactions (such as hydrogen bonds, water bridges, hydrophobic interactions, etc.) with compound 34 throughout the simulation, reflecting the contribution of the residues to maintaining ligand binding. The results showed that multiple residues had high-frequency stable interactions with compound **34**. Among them, the interaction between GLY143 and compound **34** was the most significant, with a frequency close to 0.8, indicating that it almost continuously maintained contact with compound **34** throughout the simulation. In addition, HIS41, SER144, CYS145, and other residues also showed higher interaction frequencies (all above 0.3). The interaction types of different amino acids were slightly different. For example, GLY143 mainly stabilised the binding through hydrogen bonds, while HIS41 bound through multiple types of interactions, including hydrogen bonds, water bridges, and hydrophobic interactions. These residues together contributed to the stable binding of compound **34** in the binding pocket.

To further evaluate the binding stability of the ligand in the binding pocket, we performed a time trajectory analysis of the non-covalent interactions between the protein and compound **34** during the 100 ns molecular dynamics simulation. As can be seen from Figure. 10D, these residues such as ASN142, GLY143, SER144, and CYS145 showed extensive and continuous contact with compound **34** throughout the entire simulation trajectory, with very few interruptions. This result was consistent with the interaction frequency shown in Figure. 10C and further proved the core role of these residues in maintaining ligand binding. In addition, the top curve showed the trend of the total number of contacts over time. It could be seen that, although there were fluctuations during the simulation, the total number of contacts was generally maintained at around 3, further proving the stable binding.

**Figure 11.**
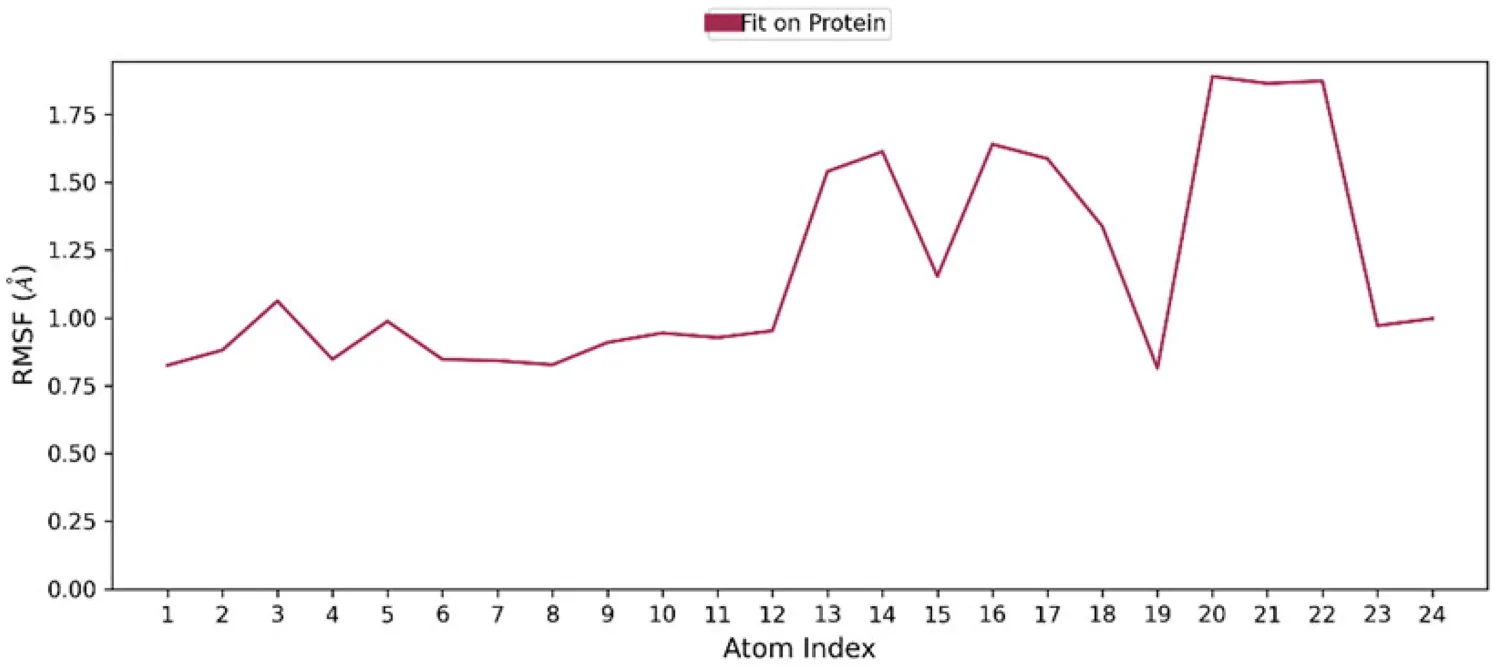
The RMSF diagram of compound **34** at the end of the 100 ns simulations.

To investigate the structural stability and flexibility distribution characteristics of compound **34** in its protein-bound state, we analysed its atomic-level RMSF during a 100 ns molecular dynamics simulation. As shown in the Figure 11, the RMSF values for most of the ligand’s atoms during the 100 ns simulation were concentrated within the range of 0.8–1.2 Å, demonstrating relatively minor fluctuations. The results also indicated that compound **34** maintained good stability throughout the binding process.

**Figure 12.**
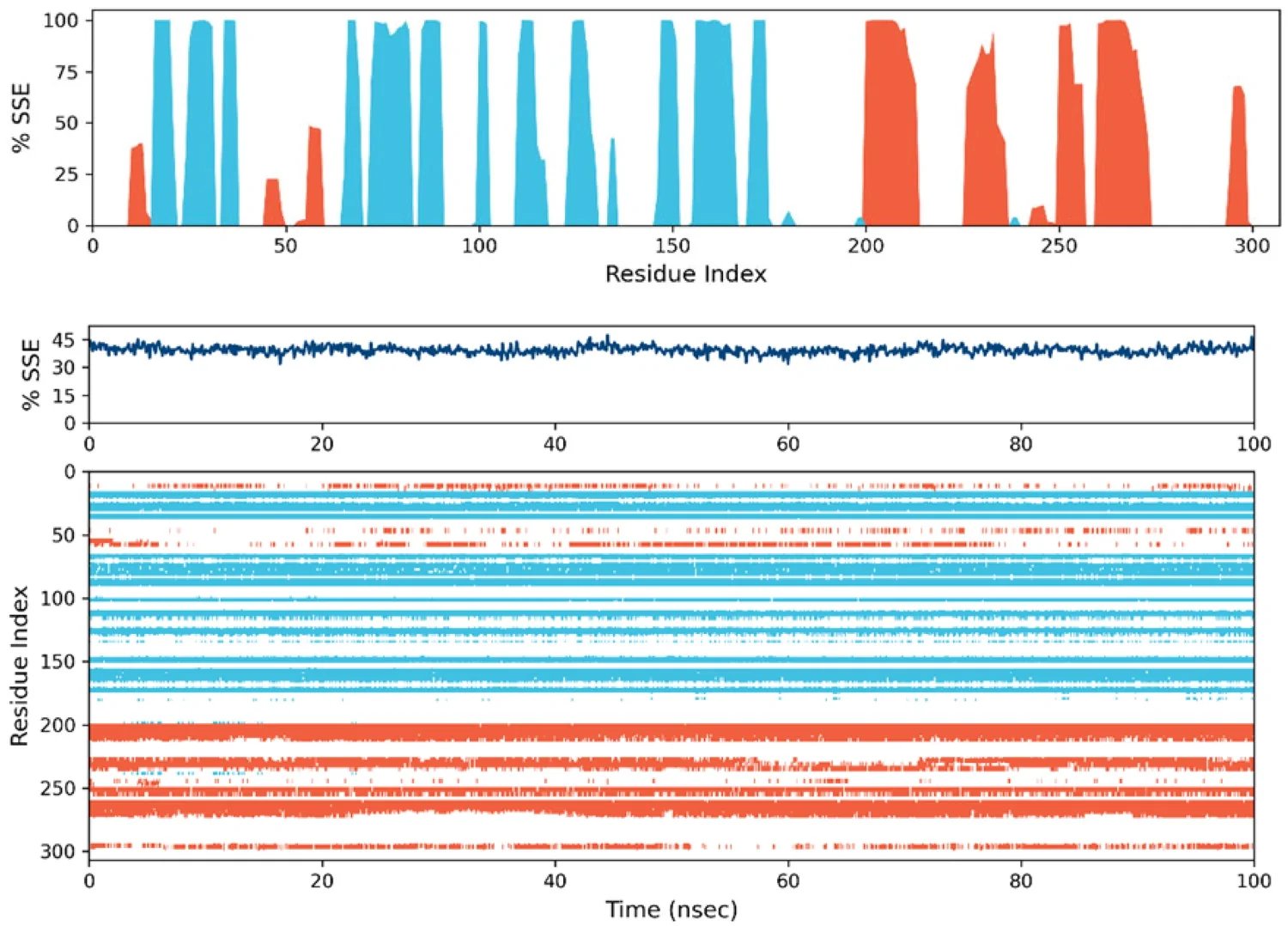
The percentage of secondary structures (α-helices or β-sheets) and their dynamic changes over the 100 ns simulation. In the structural evolution plot, blue regions represent β-sheets, while red regions represent α-helices.

Additionally, to further evaluate the conformational stability of the protein during the simulation, we characterised the percentage of secondary structures (α-helices or β-sheets) and their dynamic changes over the 100 ns simulation. In the structural evolution plot (Figure 12), blue regions represented β-sheets, while red regions represented α-helices. The analysis revealed that the dominant secondary structures were localised to residues 20–180 (β-sheets) and 190–270 (α-helices), with high retention rates throughout the simulation. Notably, the overall secondary structure content remained stable at 40–45%, and no significant disruptions or rearrangements were observed in the structural evolution map. These results indicated that the protein maintained high conformational stability during the simulation, with well-preserved secondary structures. The absence of significant structural perturbations upon compound **34** binding further underscored the robust stability of the complex.

In summary, through a systematic analysis of the 100 ns molecular dynamics simulation trajectory from four dimensions—RMSD, RMSF, key residue interaction frequency, and contact persistence, we have fully validated the structural stability and binding reliability of the SARS-CoV-2 3CL^pro^ and compound **34** complex system. The RMSD results indicated that SARS-CoV-2 3CL^pro^ and compound **34** did not undergo significant conformational rearrangements throughout the simulation, maintaining a low deviation range and demonstrating good conformational stability. The RMSF analysis further showed that the residues around the binding site had small fluctuation amplitudes, which helped the ligand to bind stably. The interaction frequency and contact time plots clearly depicted the central role of key residues (GLY143, SER144 and CYS145) in maintaining the binding, and the synergistic action of multiple sites enhanced the binding strength and persistence. These analysis results corroborated each other and collectively demonstrated that compound **34** could achieve stable and durable binding in the binding pocket.

## Conclusion

We have tested the inhibition activities of 41 novel isatin derivatives against SARS-CoV-2 3CL^pro^. The results showed that 4-trifluoromethylphenyl group at N-1 position was essential for the high inhibition activity, and superior to other hydrophobic aromatic substituents like biphenyl, naphthyl, quinolyl and indolyl groups. Additionally, trifluoromethyl group was also superior to other substituents at the 4-position of benzyl group. Furthermore, introduction of electron withdrawing groups at the C-5 and C-7 of isatin ring, such as bromo group, might be more favourable for the inhibition activity. The most potent compound **34** demonstrated an IC_50_ of 0.363 ± 0.045 µM against SARS-CoV-2 3CL^pro^, which was found to be equipotent to the positive drug Ebselen. This remarkable observation was supported by molecular docking and molecular dynamic simulation, which showed the interaction of compound **34** with several important amino acid residues in the binding site of SARS-CoV-2 3CL^pro^ and the stability of the formed interactions between the compound and the active site. The binding of compound **34** to SARS-CoV-2 3CL^pro^ was confirmed by SPR using a two-state kinetics model, with a measured K_D_ of 2.152 × 1 0 –6 M. For compound **34**, inhibition of the enzyme was reversible within 2 h. Thereafter, their antiviral activities were demonstrated by their capacities to inhibit the proliferation of viral cells. Remarkably, compound **34** exerted promising cell growth inhibitory activity with IC_50_=0.402 ± 0.021 *μ*M (SI = 170). In-silico ADME analysis showed that compound **34** possess significant drug-like characteristics based on Lipinski’s rule of 5. These results collectively exhibited that compound **34** might be chose as a novel lead compound against SARS-CoV-2 3CL^pro^ inhibitor for further study. Further optimisation to reduce the cytotoxicity and to increase the cellular activity and solubility is necessary to develop this series of compounds as SARS-CoV-2 3CL^pro^ inhibitor.

## Experimental section

### General chemistry

Melting points were determined on an XT5B microscopic melting point apparatus and were uncorrected.^1^H NMR spectra were recorded on a Varian VNMRS-600 spectrometer at 600 MHz using tetramethyl silane (TMS) as internal standard.^13^C NMR spectra were recorded on a Varian VNMRS-600 spectrometer at 151 MHz using tetramethyl silane (TMS) as internal standard. High-resolution electrospray ionisation (HR-ESI) mass spectra were recorded on a Thermo Fisher Scientific Q Exactive (Bremen, Germany) Ultra High-Resolution Mass Spectrometer. Column chromatography was carried out on silica gel (100 mesh, eluent: petroleum ether: ethyl acetate = 100:1–10:1). Purity of compound **34** was estimated by HPLC (Ultimate 3000 UPLC, UV detection at 262 nm) analysis on the column (Accucorea Q 100 × 2.1 mm, 2.6 μm, Thermo Scientific). HPLC conditions: Mobile phase methanol (A) and water(B); Gradient elution: t = 0-5-10-10-15, A%=15-95-95-15-15; Flow rate: 0.2 ml/min. The results were presented in the Supplement materials. All reactions were monitored by thin layer chromatography (TLC), carried out on silica gel 60 F-254 aluminium sheets using UV, light (254 and 366 nm). Chemicals & solvents used for the synthesis were obtained commercially and used without further purification, such as 5, 7-dibromo-1H-indole-2, 3-dione and halogen substituted isatin derivatives.

### General procedure for the synthesis of compounds 1–27

At 0 °C, 19.2 mg of sodium hydroxide (0.48 mmol) was added to a solution of isatin (0.48 mmol) in 10 ml of DMF. The mixture was stirred for 30 min, and then 0.72 mmol of RBr was added. The mixture was stirred for 30 min at room temperature, added with 30 ml of water, extracted by 50 ml of ethyl acetate, washed with 30 ml of saturated NaCl and dried over Na_2_SO_4_. After solvent removal, the crude product was purified by silica gel column chromatography with petroleum ether and ethyl acetate to afford compounds **1–27**.

#### 1-benzyl-indole-2,3-dione ^(1)^

Compound **1** was prepared according to general procedure above by using isatin and benzyl bromide. Orange powder; Yield 91%; Mp182–184 °C; ^1^H NMR (600 MHz, CDCl_3_)*δ*: 7.60 (d, *J* = 7.40 Hz, 1H), 7.48 (t, *J* = 7.73 Hz, 1H), 7.34 (d, *J* = 6.36 Hz, 4H), 7.30(ddd, *J* = 8.54, 5.44, 2.27 Hz, 1H), 7.08 (t, *J* = 7.53 Hz, 1H), 6.78 (d, *J* = 7.92 Hz, 1H), 4.93 (s, 2H); ^13^C NMR (151 MHz, CDCl_3_)*δ*: 183.35, 158.39, 150.84, 138.43, 134.62, 129.17(2 C), 128.28, 127.55(2 C), 125.52, 123.98, 117.80, 111.12, 44.16; HRMS(ESI^+^) m/z: calcd for C_15_H_11_NO_2_ [M + H]^+^ 238.0868, found 238.0862.

#### 1-(4-methylbenzyl)-indole-2,3-dione ^(2)^

Compound **2** was prepared according to general procedure above by using isatin and 4-methyl-benzyl bromide. Orange powder; Yield 89%; Mp196–198 °C; ^1^H NMR (600 MHz, CDCl_3_) *δ*: 7.59 (dd, *J* = 7.43, 1.42 Hz, 1H), 7.47 (td, *J* = 7.82, 1.38 Hz, 1H), 7.22 (d, *J* = 8.04 Hz, 2H), 7.15 (d, *J* = 7.81 Hz, 2H), 7.07 (t, *J* = 7.51 Hz, 1H), 6.79 (d, *J* = 7.90 Hz, 1H), 4.88 (s, 2H), 2.32 (s, 3H); ^13^C NMR (151 MHz, CDCl_3_) *δ*: 183.45, 158.35, 150.89, 138.40, 138.08, 131.56, 129.81(2 C), 127.57(2 C), 125.45, 123.90, 117.78, 111.16, 43.92, 21.22; HRMS (ESI) m/z: calcd for C_16_H_13_NO_2_ [M + Na]^+^ 274.0844, found 274.0830.

#### 1-(4-trifluoromethylbenzyl)-indole-2, 3-dione ^(3)^

Compound **3** was prepared according to general procedure above by using isatin and 4-trifluoromethylbenzyl bromide. Orange powder; Yield 88%; Mp193–195 °C; ^1^H NMR (600 MHz, CDCl_3_) *δ*: 7.63 (ddd, *J* = 10.22, 7.74, 4.74 Hz, 3H), 7.52 (td, *J* = 7.79, 1.20 Hz, 1H), 7.47 (d, *J* = 8.04 Hz, 2H), 7.13 (td, *J* = 7.55, 2.85 Hz, 1H), 6.75 (dd, *J* = 7.97, 3.05 Hz, 1H), 5.00 (s, 2H); ^13^C NMR (151 MHz, CDCl_3_) *δ*: 182.91, 158.39, 150.38, 138.58, 130.64(m, 1 C), 127.81(2 C), 126.20, 125.76, 124.87, 124.31(2 C), 123.06, 117.83, 110.82, 43.69; HRMS (ESI) m/z: calcd for C_16_H_10_F_3_NO_2_ [M + H]^+^306.0742, found: 306.0728.

#### 1-(2,4,6-trimethylbenzyl)-indole-2,3-dione ^(4)^

Compound **4** was prepared according to general procedure above by using isatin and 2, 4, 6-trimethylbenzyl bromide. Orange powder; Yield 90%; Mp223–225 °C; ^1^H NMR (600 MHz, CDCl_3_) *δ*: 7.55 (d, *J* = 7.42 Hz, 1H), 7.34 (t, *J* = 7.83 Hz, 1H), 7.01 (t, *J* = 7.50 Hz, 1H), 6.88 (s, 2H), 6.40 (d, *J* = 8.05 Hz, 1H), 4.98 (s, 2H), 2.33 (s, 6H), 2.27 (s, 3H); ^13^C NMR (151 MHz, CDCl_3_)*δ*: 183.44, 158.12, 151.19, 138.47, 138.05, 137.31(2 C), 130.07(2 C), 126.95, 125.29, 123.60, 117.87, 111.52, 39.92, 20.99, 20.54(2 C); HRMS(ESI^+^)m/z: calcd for C_18_H_17_NO_2_ [M + Na]^+^302.1157, found 302.1145.

#### 1-(4-isopropylbenzyl)-indole-2,3-dione ^(5)^

Compound **5** was prepared according to general procedure above by using isatin and 4-isopropylbenzyl bromide. Orange powder; Yield 89%; Mp 192–194 °C; ^1^H NMR (600 MHz, CDCl_3_) *δ*: 7.59 (d, *J* = 7.43 Hz, 1H), 7.49 (t, *J* = 7.78 Hz, 1H), 7.26 (m, 2H), 7.20 (d, *J* = 8.14 Hz, 2H), 7.08 (t, *J* = 7.50 Hz, 1H), 6.82 (d, *J* = 7.97 Hz, 1H), 4.89 (s, 2H), 2.88 (hept, *J* = 6.89 Hz, 1H), 1.22(d, *J* = 6.93 Hz, 6H); ^13^C NMR (151 MHz, CDCl_3_) *δ*: 183.48, 158.36, 150.97, 149.04, 138.41, 131.94, 127.65(2 C), 127.20(2 C), 125.46, 123.89, 117.79, 111.16, 43.92, 33.90, 24.01(2 C); HRMS(ESI^+^)m/z: calcd for C_18_H_17_NO_2_ [M + Na]^+^ 302.1157, found 302.1143.

#### 1-(4-trifluoromethoxybenzyl)-indole-2,3-dione ^(6)^

Compound **6** was prepared according to general procedure above by using isatin and 4-trifluoromethoxybenzyl bromide. Orange powder; Yield 87%; Mp 214–216 °C; ^1^H NMR (600 MHz, CDCl_3_) *δ*: 7.63 (d, *J* = 7.42 Hz, 1H), 7.52 (td, *J* = 7.80, 1.37 Hz, 1H), 7.38 (d, *J* = 8.59 Hz, 2H), 7.20(d, *J* = 8.19 Hz, 2H), 7.12 (t, *J* = 7.53 Hz, 1H), 6.77 (d, *J* = 7.95 Hz, 1H), 4.93 (s, 2H); ^13^C NMR (151 MHz, CDCl_3_) *δ*: 183.03, 158.37, 150.51, 149.16, 138.53, 133.41, 129.08(2 C), 125.76, 124.24, 121.69, 121.37, 119.66, 117.86, 110.85, 43.46; HRMS(ESI^+^)m/z: calcd for C_16_H_10_F_3_NO_3_ [M + Na]^+^344.0510, found 344.0495.

#### 1-(4-nitrobenzyl)-indole-2,3-dione ^(7)^

Compound **7** was prepared according to general procedure above by using isatin and 4-nitrobenzyl bromide. Orange powder; Yield 89%; Mp224–226 °C; ^1^H NMR (600 MHz, CDCl_3_)*δ*: 8.22 (d, *J* = 8.67 Hz, 2H), 7.66 (dd, *J* = 7.47, 1.31 Hz, 1H), 7.52 (m, 3H), 7.15 (t, *J* = 7.53 Hz, 1H), 6.72 (d, *J* = 7.96 Hz, 1H), 5.04 (s, 2H); ^13^C NMR (151 MHz, CDCl_3_)*δ*: 182.59, 158.36, 150.11, 147.99, 141.98, 138.62, 128.31(2 C), 125.97, 124.54, 124.49(2 C), 117.90, 110.63, 43.56; HRMS(ESI^+^)m/z: calcd for C_15_H_10_N_2_O_4_ [M + Na]^+^ 305.0538, found 305.0525.

#### 1-(4-cyanobenzyl)-indole-2,3-dione ^(8)^

Compound **8** was prepared according to general procedure above by using isatin and 4-cyanobenzyl bromide. Orange powder; Yield 92%; Mp 279–280 °C; ^1^H NMR (600 MHz, CDCl_3_)*δ*: 7.65 (m, 3H), 7.52 (td, *J* = 7.79, 1.28 Hz, 1H), 7.45 (d, *J* = 8.10 Hz, 2H), 7.14 (d, *J* = 7.56 Hz, 1H), 6.70 (d, *J* = 7.88 Hz, 1H), 4.99(s, 2H); ^13^C NMR (151 MHz, CDCl_3_)*δ*: 182.65, 158.36, 150.18, 140.04, 138.60, 133.04(2 C), 128.15(2 C), 125.92, 124.48, 118.36, 117.87, 112.47, 110.65, 43.77; HRMS(ESI^+^)m/z: calcd for C_16_H_10_N_2_O_2_ [M + Na]^+^ 285.0640, found 285.0626.

#### 1-(4-carbomethoxybenzyl)-indole-2,3-dione ^(9)^

Compound **9** was prepared according to general procedure above by using isatin and 4-carbomethoxybenzyl bromide. Orange powder; Yield 86%; Mp 185–187 °C; ^1^H NMR (600 MHz, CDCl_3_)*δ*: 8.01 (s, 1H), 7.98 (d, *J* = 7.76 Hz, 1H), 7.62 (d, *J* = 7.43 Hz, 1H), 7.53 (d, *J* = 7.92 Hz, 1H), 7.48 (td, *J* = 7.83, 1.20 Hz, 1H), 7.43 (t, *J* = 7.71 Hz, 1H), 7.10 (t, *J* = 7.52 Hz, 1H), 6.75 (d, *J* = 7.96 Hz, 1H), 4.97 (s, 2H), 3.91 (s, 3H); ^13^C NMR (151 MHz, CDCl_3_) *δ*: 183.09, 166.65, 158.41, 150.54, 138.52, 135.20, 131.98, 131.13, 129.59, 129.44, 128.62, 125.69, 124.18, 117.85, 110.95, 52.43, 43.90; HRMS(ESI^+^): m/z: calcd for C_17_H_13_NO_4_ [M + H]^+^ 296.0923, found 296.0911.

#### 1-(4-fluorobenzyl)-indole-2,3-dione ^(10)^

Compound **10** was prepared according to general procedure above by using isatin and 4-fluorobenzyl bromide. Orange powder; Yield 85%; Mp 222–224 °C;

^1^H NMR (600 MHz, CDCl_3_)*δ*: 7.60 (d, *J* = 7.44 Hz, 1H), 7.50 (td, *J* = 7.82, 1.25 Hz, 1H), 7.32(dd, *J* = 8.56, 5.24 Hz, 2H), 7.10 (t, *J* = 7.54 Hz, 1H), 7.02 (t, *J* = 8.46 Hz, 2H), 6.78 (d, *J* = 7.96 Hz, 1H), 4.89 (s, 2H); ^13^C NMR (151 MHz, CDCl_3_) *δ*: 183.18, 162.58(d, *J* = 247.37 Hz, 1 C), 158.33, 150.58, 138.46, 130.47, 129.40, 129.34, 125.61, 124.09, 117.79, 116.20, 116.05, 110.93, 43.45; HRMS(ESI^+^)m/z: calcd for C_15_H_10_FNO_2_ [M + H]^+^256.0774, found 256.0762.

#### 1-(2, 3-difluorobenzyl)-indole-2, 3-dione ^(11)^

Compound **11** was prepared according to general procedure above by using isatin and 2, 3-difluorobenzyl bromide. Orange powder; Yield 88%; Mp194–196 °C; ^1^H NMR (600 MHz, CDCl_3_) *δ*: 7.62 (d, *J* = 7.55 Hz, 1H), 7.55 (t, *J* = 7.81 Hz, 1H), 7.13(m, 3H), 7.06 (tdd, *J* = 8.51, 4.71, 1.49 Hz, 1H), 6.89 (d, *J* = 7.95 Hz, 1H), 5.00 (s, 2H); ^13^C NMR (151 MHz, CDCl_3_)*δ*:182.87, 158.42, 150.26, 138.71, 125.70(2 C), 124.99(dd, J = 6.80, 4.63 Hz, 1 C), 124.66, 124.28(2 C), 117.81, 117.51, 117.40, 110.62, 37.10; HRMS(ESI^+^)m/z: calcd for C_15_H_9_F_2_NO_2_ [M + H]^+^ 274. 0680, found 274.0667.

#### 1-(2,4,5-trifluorobenzyl)-indole-2,3-dione ^(12)^

Compound **12** was prepared according to general procedure above by using isatin and 2, 4, 5-trifluorobenzyl bromide. Orange powder; Yield 92%; Mp 166– 168 °C; ^1^H NMR (600 MHz, CDCl_3_) *δ*: 7.63 (d, *J* = 7.43 Hz, 1H), 7.56 (t, *J* = 7.81 Hz, 1H), 7.22 (m, 1H), 7.14 (t, *J* = 7.54 Hz, 1H), 6.98 (td, *J* = 9.58, 6.40 Hz, 1H), 6.88 (d, *J* = 7.97 Hz, 1H), 4.91(s, 2H); ^13^C NMR (151 MHz, CDCl_3_) *δ*: 182.67, 158.47, 150.00, 138.73(2 C), 125.83(2 C), 124.43(2 C), 117.83, 110.46, 106.27, 106.10(d, *J* = 6.77 Hz, 1 C), 105.94, 36.82; HRMS(ESI^+^)m/z: calcd for C_15_H_8_F_3_NO_2_ [M + H]^+^ 292.0585, found 292.0574.

#### 1-(4-chlorobenzyl) -indole-2,3-dione ^(13)^

Compound **13** was prepared according to general procedure above by using isatin and 4-chlorobenzyl bromide. Orange powder; Yield 92%; Mp 224–226 °C; ^1^H NMR (600 MHz, CDCl_3_) *δ*: 7.61 (dd, *J* = 7.46, 1.26 Hz, 1H), 7.51(td, *J* = 7.80, 1.36 Hz, 1H), 7.30 (m, 4H), 7.11 (t, *J* = 7.53 Hz, 1H), 6.77 (d, *J* = 7.87 Hz, 1H), 4.90 (s, 2H); ^13^C NMR (151 MHz, CDCl_3_)*δ*: 183.07, 158.32, 150.47, 138.48, 134.16, 133.16, 129.33(2 C), 128.92(2 C), 125.61, 124.14, 117.76, 110.92, 43.48; HRMS(ESI^+^) m/z: calcd for C_15_H_10_ClNO_2_ [M + Na]^+^ 294.0298, found 294.0285.

#### 1-(3, 4-dichlorobenzyl)-indole-2, 3-dione ^(14)^

Compound **14** was prepared according to general procedure above by using isatin and 3, 4-dichlorobenzyl bromide. Orange powder; Yield 93%; Mp249–251 °C; ^1^H NMR (600 MHz, CDCl_3_)*δ*: 7.64 (d, *J* = 7.44 Hz, 1H), 7.53(td, *J* = 7.84, 1.23 Hz, 1H), 7.42 (d, *J* = 6.43 Hz, 2H), 7.18 (dd, *J* = 8.27, 2.04 Hz, 1H), 7.13 (t, *J* = 7.54 Hz, 1H), 6.75 (d, *J* = 7.95 Hz, 1H), 4.88 (s, 2H); ^13^C NMR (151 MHz, CDCl_3_) *δ*: 182.80, 158.33, 150.25, 138.59, 134.94, 133.43, 132.65, 131.23, 129.49, 126.87, 125.84, 124.38, 117.86, 110.76, 43.13; HRMS(ESI^+^)m/z: calcd for C_15_H_9_Cl_2_NO_2_ [M + Na]^+^ 327.9908, found 327.9893.

#### 1- (2, 4-dichlorobenzyl)-indole-2, 3-dione ^(15)^

Compound **15** was prepared according to general procedure above by using isatin and 2, 4-dichlorobenzyl bromide. Orange powder; Yield 92%; Mp 248–250 °C; ^1^H NMR (600 MHz, CDCl_3_)*δ*: 7.64 (d, *J* = 7.44 Hz, 1H), 7.52 (td, *J* = 7.78, 1.32 Hz, 1H), 7.44 (d, *J* = 1.92 Hz, 1H), 7.20 (dd, *J* = 8.37, 2.01 Hz, 1H), 7.17 (d, *J* = 8.41 Hz, 1H), 7.14 (t, *J* = 7.54 Hz, 1H), 6.75 (d, *J* = 7.90 Hz, 1H), 5.01 (s, 2H); ^13^C NMR (151 MHz, CDCl_3_) *δ*:182.82, 158.52, 150.31, 138.70, 134.77, 133.78, 130.64, 129.91, 129.35, 127.95, 125.74, 124.38, 117.84, 110.98, 41.12; HRMS(ESI^+^)m/z: calcd for C_15_H_9_Cl_2_NO_2_ [M + H]^+^306.0089, found 306.0078.

#### 1-(4-bromobenzyl)-indole-2,3-dione ^(16)^

Compound **16** was prepared according to general procedure above by using isatin and 4-bromobenzyl bromide. Orange powder; Yield 87%; Mp: 245–247 °C; ^1^H NMR (600 MHz, CDCl_3_) *δ*: 7.61 (dd, *J* = 7.43, 1.30 Hz, 1H), 7.49 (td, *J* = 7.91, 1.19 Hz, 1H), 7.47 (d, *J* = 8.44 Hz, 2H), 7.21 (d, *J* = 8.42 Hz, 2H), 7.10 (t, *J* = 7.53 Hz, 1H), 6.74 (d, *J* = 7.93 Hz, 1H), 4.87(s, 2H); ^13^C NMR (151 MHz, CDCl_3_) *δ*: 183.05, 158.33, 150.48, 138.49, 133.69, 132.33(2 C), 129.25(2 C), 125.66, 124.18, 122.29, 117.80, 110.92, 43.57; HRMS(ESI^+^)m/z: calcd for C_15_H_10_BrNO_2_ [M + H]^+^ 315.9973, found 315.9960.

#### 1-(8-quinolylmethyl)-indole-2, 3-dione ^(17)^

Compound **17** was prepared according to general procedure above by using isatin and 8-quinolylmethyl bromide. Orange powder; Yield 88%; Mp235–237 °C; ^1^H NMR (600 MHz, CDCl_3_) δ: 9.00 (dd, J = 4.20, 1.80 Hz, 1H), 8.20 (dd, J = 8.32, 1.78 Hz, 1H), 7.78 (dd, J = 8.22, 1.33 Hz, 1H), 7.69 (dd, J = 7.12, 1.27 Hz, 1H), 7.59 (dd, J = 7.42, 1.31 Hz, 1H), 7.48 (dd, J = 8.31, 1.98 Hz, 2H), 7.41 (td, J = 7.81, 1.39 Hz, 1H), 7.04 (td, J = 7.54, 0.84 Hz, 1H), 7.02 (d, J = 8.00 Hz, 1H), 5.69 (s, 2H); ^13^C NMR (151 MHz, CDCl_3_) δ: 183.75, 158.99, 151.33, 149.96, 146.11, 138.48, 136.64, 132.97, 128.49, 128.42, 128.16, 126.63, 125.26, 123.80, 121.61, 117.79, 111.82, 39.57; HRMS(ESI^+^) m/z: calcd for C_18_H_12_N_2_O_2_ [M + H]^+^ 289.0977, found 289.0965.

#### 1-(4-phenylbenzyl) -indole-2, 3-dione ^(18)^

Compound **18** was prepared according to general procedure above by using isatin and 4-phenylbenzyl bromide. Orange powder; Yield 88%; Mp 239–241 °C; ^1^H NMR (600 MHz, CDCl_3_) δ: 7.62(d, J = 7.39 Hz, 1H), 7.56(d, J = 11.27 Hz, 4H), 7.50(t, J = 7.79 Hz, 1H), 7.42(m, 4H), 7.35 (t, J = 7.31 Hz, 1H), 7.10 (t, J = 7.53 Hz,1H), 6.83(d, J = 7.97 Hz, 1H), 4.97 (s, 2H); ^13^C NMR (151 MHz, CDCl_3_) δ: 183.35, 158.42, 150.83, 141.28, 140.44, 138.48, 133.59, 128.97(2 C), 128.05(2 C), 127.87(2 C), 127.68, 127.16(2 C), 125.58, 124.03, 117.84, 111.15, 43.91; HRMS(ESI^+^)m/z: calcd for C_21_H_15_NO_2_ [M + Na]^+^336.1000, found 336.0985.

#### 1-(1-carboxylic acid tert-butyl ester-indole-3-methyl)-indole-2, 3-dione ^(19)^

Compound **19** was prepared according to general procedure above by using isatin and 1-carboxylic acid tert-butyl ester-indole-3-methyl bromide. Orange powder; Yield 87%; Mp159–161 °C; ^1^H NMR (600 MHz, CDCl_3_) δ: 8.08(d, J = 8.25 Hz, 1H), 7.66 (m, 2H), 7.58 (d, J = 7.42 Hz, 1H), 7.51 (td, J = 7.83, 1.23 Hz, 1H), 7.33 (td, J = 7.47, 0.89 Hz, 1H), 7.24 (d, J = 7.53 Hz, 1H), 7.08 (t, J = 7.50 Hz, 1H), 6.99 (d, J = 7.99 Hz, 1H), 5.04 (s, 2H), 1.67 (s, 9H); ^13^C NMR (151 MHz, CDCl_3_) δ: 183.25, 158.18, 150.79, 149.65, 138.41, 135.59, 128.84, 125.50, 125.21, 125.18, 123.97, 123.25, 119.51, 117.83, 115.50, 114.17, 111.05, 84.51, 35.78, 28.30(3 C); HRMS(ESI^+^):m/z calcd for C_22_H_20_N_2_O_4_ [M + Na]^+^ 399.1321, found 399.1316.

#### 1-allyl-indole-2, 3-dione ^(20)^

Compound **20** was prepared according to general procedure above by using isatin and allyl bromide. Orange powder; Yield 89%; Mp158–160 °C; ^1^H NMR (600 MHz, CDCl_3_) δ: 7.60 (d, J = 7.06 Hz, 1H), 7.56 (t, J = 7.79 Hz, 1H), 7.11 (t, J = 7.52 Hz, 1H), 6.89 (d, J = 7.84 Hz, 1H), 5.84(ddd, J = 22.53, 10.52, 5.35 Hz, 1H), 5.30 (m, 2H), 4.36 (d, J = 5.47 Hz, 2H); ^13^CNMR (151 MHz, CD_3_OD) δ: 183.35, 158.01, 150.94, 138.44, 130.45, 125.48, 123.91, 118.75, 117.68, 111.01, 42.61; HRMS(ESI^+^)m/z: calcd for C_11_H_9_NO_2_ [M + H]^+^ 188.0712, found 188.0701.

#### 1-(1-naphthylmethyl)-indole-2,3-dione ^(21)^

Compound **21** was prepared according to general procedure above by using isatin and 1-naphthylmethyl bromide. Orange powder; Yield 86%; Mp229–231 °C; ^1^H NMR (600 MHz, CDCl_3_) δ: 8.08 (d, J = 8.32 Hz, 1H), 7.90 (d, J = 8.04 Hz, 1H), 7.83 (d, J = 7.31 Hz, 1H), 7.60 (m, 2H), 7.55 (t, J = 7.49 Hz, 1H), 7.41 (m, 3H), 7.07 (d, J = 7.51 Hz, 1H), 6.74 (d, J = 8.01 Hz, 1H), 5.41 (s, 2H); ^13^C NMR (151 MHz, CDCl_3_) δ: 183.21, 158.49, 151.21, 138.48, 134.04, 131.01, 129.39, 129.19, 129.04, 127.05, 126.37, 125.48, 125.34, 125.13, 124.01, 122.84, 117.90, 111.58, 42.50; HRMS(ESI^+^) m/z: calcd for C_19_H_13_NO_2_ [M + Na]^+^310.0844, found 310.0828.

#### 1-(1-bromo-2-naphthylmethyl) -indole-2, 3-dione ^(22)^

Compound **22** was prepared according to general procedure above by using isatin and 1-bromo-2-naphthylmethyl bromide. Yellow powder; Yield 87%; Mp 294–296 °C; ^1^H NMR (600 MHz, CDCl_3_) δ: 8.36 (d, J = 8.59 Hz, 1H), 7.81 (d, J = 8.08 Hz, 1H), 7.75 (d, J = 8.51 Hz, 1H), 7.65(ddd, J = 8.48, 6.90, 1.25 Hz, 2H), 7.55 (t, J = 7.48 Hz, 1H), 7.44 (t, J = 7.80 Hz, 1H), 7.29 (d, J = 8.51 Hz, 1H), 7.10 (t, J = 7.76 Hz, 1H), 6.77 (d, J = 7.99 Hz, 1H), 5.31 (s, 2H); ^13^C NMR (151 MHz, CDCl_3_) δ: 183.15, 158.66, 150.62, 138.70, 134.24, 132.46, 131.66, 128.77, 128.41, 128.16, 127.36, 127.22, 125.59, 124.78, 124.25, 123.39, 117.87, 111.43, 45.12. HRMS(ESI^+^)m/z: calcd for C_19_H_12_BrNO_2_ [M + Na]^+^ 387.9949, found 387.9934.

#### 1-(1-bromo-4-naphthylmethyl)-indole-2, 3-dione ^(23)^

Compound **23** was prepared according to general procedure above by using isatin and 1-bromo-4-naphthylmethyl bromide. Yellow powder; Yield 87%; Mp247– 249 °C; ^1^H NMR (600 MHz, CDCl_3_)δ: 8.32 (d, J = 9.73 Hz, 1H), 8.06 (d, J = 9.52 Hz, 1H), 7.70 (d, J = 7.67 Hz, 1H), 7.65 (m, 2H), 7.62 (d, J = 7.40 Hz, 1H), 7.42 (t, J = 7.78 Hz, 1H), 7.23 (d, J = 7.68 Hz, 1H), 7.08 (t, J = 7.53 Hz, 1H), 6.71 (d, J = 7.99 Hz, 1H), 5.35 (s, 2H); ^13^C NMR (151 MHz, CDCl_3_) δ: 182.94, 158.45, 150.90, 138.53, 132.38, 132.10, 129.55, 129.41, 128.47, 127.90, 127.81, 125.60, 125.39, 124.18, 123.79, 123.27, 117.88, 111.42, 42.28; HRMS(ESI^+^)m/z: calcd for C_19_H_12_BrNO_2_ [M + Na]^+^387.9949, found 387.9938.

#### 1-(3-phenylbenzyl) -indole-2,3-dione ^(24)^

Compound **24** was prepared according to general procedure above by using isatin and 3-phenylbenzyl bromide. Orange and yellow powder; Yield 87%; Mp 203–205 °C; ^1^H NMR (600 MHz, CDCl_3_) δ: 7.61(d, J = 7.33 Hz, 1H), 7.55(m, 4H), 7.49 (t, J = 7.80 Hz, 1H), 7.43(m, 3H), 7.36(t, J = 7.37 Hz, 1H), 7.32(d, J = 7.68 Hz, 1H), 7.09(t, J = 7.51 Hz, 1H), 6.82(d, J = 7.96 Hz, 1H), 4.99 (s, 2H); ^13^C NMR (151 MHz, CDCl_3_) δ: 183.31,158.44, 150.82, 142.26, 140.54, 138.48, 135.23, 129.62, 128.99(2 C), 127.79, 127.29(2 C), 127.15, 126.40, 126.32, 125.58, 124.03, 117.83, 111.11, 44.25; HRMS(ESI^+^)m/z: calcd for C_21_H_15_NO_2_ [M + H]^+^314.1181, found 314.1169.

#### 1-(2-quinolylmethyl) -indole-2,3-dione ^(25)^

Compound **25** was prepared according to general procedure above by using isatin and 2-quinolylmethyl bromide. Orange and yellow powder; Yield 86%; Mp 213–215 °C; ^1^H NMR (600 MHz, CDCl_3_) δ: 8.14 (d, J = 8.41 Hz, 1H), 8.07(d, J = 8.48 Hz, 1H), 7.80 (d, J = 8.05 Hz, 1H), 7.73 (t, J = 7.69 Hz, 1H), 7.60 (d, J = 7.40 Hz, 1H), 7.55 (t, J = 7.47 Hz, 1H), 7.46 (t, J = 7.81 Hz, 1H), 7.42 (d, J = 8.47 Hz, 1H), 7.07 (t, J = 7.53 Hz, 1H), 7.02 (d, J = 8.00 Hz, 1H), 5.22 (s, 2H); ^13^C NMR (151 MHz, CDCl_3_) δ: 183.25, 158.46, 154.94, 150.94, 147.69, 138.57, 137.85, 130.17, 129.20, 127.81, 127.66, 127.08, 125.42, 124.09, 119.63, 117.77, 111.70, 46.84; HRMS(ESI^+^)m/z: calcd for C_18_H_12_N_2_O_2_ [M + H] ^+^ 289.0977, found 289.0963.

#### 1-(4–(2-cyanophenyl)-benzyl)-indole-2,3-dione ^(26)^

Compound **26** was prepared according to general procedure above by using isatin and 4–(2-cyanophenyl)-benzyl bromide. Orange and yellow powder; Yield 90%; Mp 254–256 °C; ^1^H NMR (600 MHz, CDCl_3_) δ: 7.75 (d, J = 7.75 Hz, 1H), 7.64 (m, 2H), 7.55 (d, J = 8.21 Hz, 2H), 7.52 (d, J = 7.78 Hz, 1H), 7.47 (m, 4H), 7.12 (t, J = 7.53 Hz, 1H), 6.84 (d, J = 7.97 Hz, 1H), 4.99 (s, 2H); ^13^C NMR (151 MHz, CDCl_3_) δ:183.21, 158.44, 150.72, 144.73, 138.61, 138.18, 135.28, 133.94, 133.07, 130.15, 129.59(2 C), 127.95, 127.89(2 C), 125.65, 124.15, 118.71, 117.84, 111.28, 111.08, 43.84. HRMS(ESI^+^) m/z: calcd for C_22_H_14_N_2_O_2_ [M + H]^+^ 339.1134, found 339.1120.

#### 1-(4-(2-carbomethoxyphenyl)-benzyl)-indole-2,3-dione ^(27)^

Compound **27** was prepared according to general procedure above by using isatin and 4-(2-carbomethoxyphenyl)-benzyl bromide. Orange powder; Yield 88%; Mp227-229 °C; ^1^H NMR (600 MHz, CDCl_3_) δ: 7.83(d, J = 7.71 Hz, 1H), 7.63(d, J = 7.38 Hz, 1H), 7.51(q, J = 7.34 Hz, 2H), 7.41(t, J = 7.31 Hz, 1H), 7.37(d, J = 8.06 Hz, 2H), 7.32(d, J = 7.52 Hz, 1H), 7.29(d, J = 8.10 Hz, 2H), 7.11(t, J = 7.53 Hz, 1H), 6.84(d, J = 7.96 Hz, 1H), 4.98 (s, 2H), 3.61 (s, 3H); ^13^C NMR (151 MHz, CDCl_3_) δ: 183.34, 168.90, 158.41, 150.90, 141.91, 141.48, 138.40, 133.53, 131.52, 130.82, 130.73, 130.09, 129.17(2 C), 127.56, 127.28(2 C), 125.59, 124.04, 117.87, 111.13, 52.06, 44.00; HRMS(ESI^+^)m/z: calcd for C_23_H_17_N_2_O_4_ [M + Na]^+^394.1055, found 394.1041.

### General procedure for the synthesis of compounds 28–36

At 0 °C, 19.2 mg of sodium hydroxide (0.48 mmol) was added to a solution of halogen substituted isatin derivatives (0.48 mmol) in 10 ml of DMF. The mixture was stirred for 30 min, and then 1-(bromomethyl)-4-(trifluoromethyl) benzene(172.10 mg, 0.72 mmol) was added. The mixture was stirred for 30 min at room temperature, added with 30 ml of water, extracted by 50 ml of ethyl acetate, washed with 30 ml of saturated NaCl and dried over Na_2_SO_4_. After solvent removal, the crude product was purified by silica gel column chromatography with petroleum ether and ethyl acetate to afford compounds **28–36**.

#### 5-fluoro-1-(4-(trifluoromethyl) benzyl)-indole-2, 3-dione ^(28)^

Compound **28** was prepared according to general procedure above by using 5- fluoro-1H-indole-2, 3-dione and1-(bromomethyl)-4-(trifluoromethyl)benzene. Orange powder; Yield 89%; Mp 129–131 °C; ^1^H NMR (600 MHz, CDCl_3_) *δ*: 7.60 (d, *J* = 8.18 Hz, 2H), 7.44 (d, *J* = 8.08 Hz, 2H), 7.31 (dd, *J* = 6.45, 2.68 Hz, 1H), 7.21 (td, *J* = 8.61, 2.72 Hz, 1H), 6.70 (dd, *J* = 8.61, 3.58 Hz, 1H), 4.98 (s, 2H). ^13^C NMR (151 MHz, CDCl_3_)*δ*: 182.34, 162.71, 160.38, 158.45(d, *J* = 90.85 Hz, 1 C), 146.38, 138.42, 130.71(q, *J* = 32.73 Hz, 1 C), 127.76(2 C), 126.21, 124.89(d, *J* = 24.22 Hz, 1 C), 122.99, 118.45, 112.76(d, *J* = 24.24 Hz, 1 C), 112.06(d, *J* = 7.22 Hz, 1 C), 43.76; HRMS(ESI^+^)m/z: calcd for C_16_H_9_F_4_NO_2_ [M + Na]^+^ 346.0467, found 346.0456.

#### 5-chloro-1-(4-(trifluoromethyl) benzyl)-indole-2,3-dione ^(29)^

Compound **29** was prepared according to general procedure above by using 5-chloro-1H-indole-2, 3-dione and 1-(bromomethyl)-4-(trifluoromethyl) benzene. Orange powder; Yield 88%; Mp 150–152 °C; ^1^H NMR (600 MHz, CDCl_3_) *δ*: 7.62 (d, *J* = 8.15 Hz, 2H), 7.60 (d, *J* = 2.14 Hz, 1H), 7.47(dd, *J* = 8.42, 2.19 Hz, 1H), 7.44 (d, *J* = 8.09 Hz, 2H), 6.69 (d, *J* = 8.42 Hz, 1H), 4.99 (s, 2H); ^13^C NMR (151 MHz, CDCl_3_) *δ*: 181.91, 157.83, 148.59, 138.28, 137.93, 130.90(d, *J* = 32.73 Hz, 1 C), 130.27, 127.80 (2 C), 126.32, 125.73, 124.81, 123.00, 118.67, 112.11, 43.85; HRMS(ESI^+^)m/z: calcd for C_16_H_9_ClF_3_NO_2_ [M + Na]^+^362.0172, found 362.0170.

#### 5-bromo-1-(4-(trifluoromethyl) benzyl)-indole-2,3-dione ^(30)^

Compound **30** was prepared according to general procedure above by using 5- bromo-1H-indole-2,3-dione and1-(bromomethyl)-4-(trifluoromethyl)benzene. Orange powder; Yield 88%; Mp158–160 °C; ^1^H NMR (600 MHz, CDCl_3_) *δ*: 7.68 (d, *J* = 2.05 Hz, 1H), 7.59 (d, *J* = 8.42 Hz, 3H), 7.44 (d, *J* = 8.06 Hz, 2H), 6.67 (d, *J* = 8.34 Hz, 1H), 4.98 (s, 2H); ^13^C NMR (151 MHz, CDCl_3_) *δ*: 181.76, 157.61, 149.00, 140.74, 138.30, 130.68 (q, *J* = 32.57 Hz, 1 C), 128.37, 127.77(2 C), 126.19, 124.77, 122.96, 118.89, 117.11, 112.57, 43.71; HRMS(ESI^+^)m/z: calcd C_16_H_9_BrF_3_NO_2_ for [M + Na]^+^ 405.9666, found 405.9664.

#### 4-chloro-1-(4-(trifluoromethyl) benzyl)-indole-2,3-dione ^(31)^

Compound **31** was prepared according to general procedure above by using 4-chloro-1H-indole-2,3-dione and 1-(bromomethyl)-4-(trifluoromethyl) benzene. Orange powder; Yield 88%; Mp 177–179 °C; ^1^H NMR (600 MHz, CDCl_3_) *δ*: 7.61 (d, *J* = 8.10 Hz, 2H), 7.44 (d, *J* = 8.09 Hz, 2H), 7.42 (t, *J* = 8.07 Hz, 1H), 7.06 (d, *J* = 8.23 Hz, 1H), 6.65 (d, *J* = 7.84 Hz, 1H), 5.00 (s, 2H); ^13^C NMR (151 MHz, CDCl_3_) *δ*: 179.75, 157.51, 151.41, 138.71, 138.39, 134.31, 130.79(q, *J* = 32.75 Hz, 1 C), 127.78 (2 C), 126.28, 125.88, 124.82, 123.01, 114.94, 109.08, 43.85; HRMS(ESI^+^)m/z: calcd for C_16_H_9_ClF_3_NO_2_ [M + Na]^+^ 362.0172, found 362.0160.

#### 6-chloro-1-(4-(trifluoromethyl) benzyl)-indole-2,3-dione ^(32)^

Compound **32** was prepared according to general procedure above by using 6- chloro-1H-indole-2,3-dione and 1-(bromomethyl)-4-(trifluoromethyl)benzene. Orange powder; Yield 86%; Mp 129–131 °C; ^1^H NMR (600 MHz, CDCl_3_) *δ*: 7.59 (d, *J* = 8.14 Hz, 2H), 7.51 (d, *J* = 7.99 Hz, 1H), 7.45 (d, *J* = 8.06 Hz, 2H), 7.05(dd, *J* = 7.99, 1.64 Hz, 1H), 6.77 (d, *J* = 1.69 Hz, 1H), 4.96 (s, 2H); ^13^C NMR (151 MHz, CDCl_3_)*δ*: 181.36, 158.27, 151.30, 144.85, 138.31, 130.51(q, *J* = 32.61 Hz, 1 C), 127.70 (2 C), 126.58, 126.13, 124.75, 124.39, 122.95, 116.01, 111.45, 43.64; HRMS (ESI^+^)m/z: calcd for C_16_H_9_ClF_3_NO_2_ [M + Na]^+^362.0172, found 362.0161.

#### 4-iodo-1-(4-(trifluoromethyl) benzyl)-indole-2,3-dione ^(33)^

Compound **33** was prepared according to general procedure above by using 4- iodo-1H-indole-2,3-dione and 1-(bromomethyl)-4-(trifluoromethyl) benzene. Orange powder; Yield 90%; Mp175–177 °C; ^1^H NMR (600 MHz, CDCl_3_)*δ*: 7.61 (d, *J* = 8.02 Hz, 2H), 7.55 (d, *J* = 8.07 Hz, 1H), 7.44 (d, *J* = 7.96 Hz, 2H), 7.14 (t, *J* = 7.95 Hz, 1H), 6.72 (d, *J* = 7.85 Hz, 1H), 4.99 (s, 2H); HRMS(ESI^+^)m/z: calcd for C_16_H_9_F_3_INO_2_ [M + Na]^+^ 453.9528, found 453.9508.

#### 5,7-dibromo-1-(4-(trifluoromethyl) benzyl)-indole-2, 3-dione ^(34)^

Compound **34** was prepared according to general procedure above by using 5, 7-dibromo-1H-indole-2, 3-dione and 1-(bromomethyl)-4-(trifluoromethyl) benzene. Orange powder; Yield 91%; Mp124–126 °C; ^1^H NMR (600 MHz, CDCl_3_) *δ*: 7.84 (d, *J* = 2.02 Hz, 1H), 7.75 (d, *J* = 1.99 Hz, 1H), 7.61 (d, *J* = 8.12 Hz, 2H), 7.36(d, *J* = 8.01 Hz, 2H), 5.46 (s, 2H); ^13^C NMR (151 MHz, CDCl_3_) *δ*: 181.01, 158.38, 146.43, 145.53, 139.97, 130.35(q, *J* = 32.23 Hz, 1 C), 127.94, 126.88(2 C), 126.08(t, *J* = 3.63 Hz, 1 C), 124.94, 123.15, 121.57, 117.73, 105.23, 44.57; HRMS(ESI^+^)m/z: calcd for C_16_H_8_Br_2_F_3_NO_2_ [M]^+^ 462.8853, found 462.88605. Purity: 99.75% by HPLC (t_R_= 7.21 min).

#### 4, 6-dichloro-1-(4-(trifluoromethyl) benzyl)-indole-2, 3-dione ^(35)^

Compound **35** was prepared according to general procedure above by using 4, 6-dichloro-1H-indole-2,3-dione and 1-(bromomethyl)-4-(trifluoromethyl) benzene. Orange powder; Yield 87%; Mp156–158 °C; ^1^H NMR (600 MHz, CDCl_3_) *δ*: 7.62 (d, *J* = 8.15 Hz, 2H), 7.44 (d, *J* = 8.03 Hz, 2H), 7.05 (d, *J* = 1.51 Hz, 1H), 6.69(d, *J* = 1.63 Hz, 1H), 4.98 (s, 2H); ^13^C NMR (151 MHz, CDCl_3_) *δ*: 178.41, 157.52, 152.00, 144.92, 137.96, 134.88, 130.83(q, *J* = 32.76 Hz, 1 C), 127.74(2C), 126.32, 125.56, 124.76, 122.95, 113.34, 109.94, 43.90; HRMS(ESI^+^) m/z: calcd for C_16_H_8_Cl_2_F_3_NO_2_ [M + Na]^+^ 395.9782, found 395.9782.

#### 4-fluoro-5-chloro-1-(4-(trifluoromethyl) benzyl)-indole-2,3-dione ^(36)^

Compound **36** was prepared according to general procedure above by using 4- fluoro-5-chloro-1H-indole-2,3-dioneand1-(bromomethyl)-4-(trifluoromethyl) benzene. Orange powder; Yield 85%; Mp152-154 °C; ^1^H NMR (600 MHz, CDCl_3_) *δ*: 7.64 (d, *J* = 8.12 Hz, 2H), 7.45 (d, *J* = 8.06 Hz, 2H), 7.42 (d, *J* = 6.67 Hz, 1H), 6.79 (d, *J* = 5.23 Hz, 1H), 4.97 (s, 2H); HRMS(ESI^+^) m/z: calcd for C_16_H_8_ClF_4_NO_2_ [M + Na]^+^ 380.0077, found 380.0070.

### General procedure for the synthesis of compounds 37–41

At 0 °C, 19.2 mg of sodium hydroxide (0.48 mmol) was added to a solution of 5,7-dibromo-1H-indole-2,3-dione (0.48 mmol) in 10 ml of DMF. The mixture was stirred for 30 min, and then 0.72 mmol of RBr was added. The mixture was stirred for 30 min at room temperature, added with 30 ml of water, extracted by 50 ml of ethyl acetate, washed with 30 ml of saturated NaCl and dried over Na_2_SO_4_. After solvent removal, the crude product was purified by silica gel column chromatography with petroleum ether and ethyl acetate to afford compounds **37–41**.

#### 5,7-dibromo-1-(4-bromo-benzyl)-indole-2,3-dione ^(37)^

Compound **37** was prepared according to general procedure above by using 5, 7-dibromo-1H-indole-2,3-dione and 4-bromo-benzyl bromide. Yellow power; Yield 86%; Mp155–157 °C; ^1^H NMR (600 MHz, CDCl_3_)*δ*: 7.81 (d, *J* = 1.99 Hz, 1H), 7.71(d, *J* = 1.96 Hz, 1H), 7.44 (d, *J* = 8.39 Hz, 2H), 7.12 (d, *J* = 8.49 Hz, 2H), 5.34 (s, 2H); ^13^C NMR (151 MHz, CDCl_3_)*δ*:181.10, 158.36, 146.50, 145.42, 134.90, 132.10(2 C), 128.33(2 C), 127.78, 121.87, 121.53, 117.52, 105.21, 44.32; HRMS(ESI^+^)m/z: calcd for C_15_H_8_Br_3_NO_2_ [M + Na]^+^ 495.7982, found 495.7988.

#### 5,7-dibromo-1-(4-phenylbenzyl)-indole-2,3-dione ^(38)^

Compound **38** was prepared according to general procedure above by using 5, 7-dibromo-1H-indole-2,3-dione and 4-phenylbenzyl bromide. Yellow power; Yield 87%; Mp145–147 °C; ^1^H NMR (600 MHz, CDCl_3_)*δ*: 7.80(d, *J* = 2.02 Hz, 1H), 7.70(d, *J* = 2.02 Hz, 1H), 7.56 (d, *J* = 8.05 Hz, 4H), 7.42 (t, *J* = 7.72 Hz, 2H), 7.35(m, 1H), 7.32 (d, *J* = 8.18 Hz, 2H), 5.43 (s, 2H); ^13^C NMR (150 MHz, CDCl_3_)*δ*: 181.32, 158.43, 146.76, 145.37, 140.74, 140.41, 134.77, 128.89(2 C), 128.87, 127.59((t, *J* = 5.88 Hz, 2 C), 127.40, 127.30, 127.09, 127.06(2 C), 121.51, 117.29, 105.32, 44.54; HRMS (ESI^+^)m/z: calcd for C_21_H_13_Br_2_NO_2_ [M + Na]^+^493.9190, found 493.9137.

#### 5,7-dibromo-1-(4-nitro-benzyl)-indole-2,3-dione ^(39)^

Compound **39** was prepared according to general procedure above by using 5, 7-dibromo-1H-indole-2,3-dione and 4-nitro-benzyl bromide. Yellow power; Yield 88%; Mp147–149 °C; ^1^H NMR (600 MHz, CDCl_3_)*δ*: 8.21(d, *J* = 8.69 Hz, 2H), 7.84(d, *J* = 2.00 Hz, 1H), 7.76(d, *J* = 1.98 Hz, 1H), 7.42(m, 2H), 5.49(s, 2H); ^13^C NMR (150 MHz, CDCl_3_)*δ*: 180.73, 158.33, 147.72, 146.13, 145.49, 143.32, 128.05, 127.35 (2 C), 124.34(2 C), 121.56, 117.94, 105.10, 44.53; HRMS(ESI^+^)m/z: calcd for C_15_H_8_Br_2_N_2_O_4_ [M]^+^439.8830, found 439.88376.

#### 5,7-dibromo-1-(4-cyano-benzyl)-indole-2,3-dione ^(40)^

Compound **40** was prepared according to general procedure above by using 5, 7-dibromo-1H-indole-2,3-dione and 4-cyano-benzylbromide. Yellow power; Yield 86%; Mp180–182 °C; ^1^H NMR (600 MHz, CDCl_3_)*δ*: 7.84 (d, *J* = 2.01 Hz, 1H), 7.76(d, *J* = 2.00 Hz, 1H), 7.65(m, 2H), 7.36(m, 2H), 5.46(s, 2H); ^13^C NMR (151 MHz, CDCl_3_) *δ*: 180.79, 158.33, 146.21, 145.52, 141.35, 132.91(2 C), 128.03, 127.25(2 C), 121.56, 118.49, 117.90, 112.10, 105.13, 44.67; HRMS(ESI^+^)m/z: calcd for C_16_H_8_Br_2_N_2_O_2_ [M + Na]^+^442.8830, found 442.8840.

#### 5,7-dibromo-1-(4–(2-cyanophenyl)-benzyl)-indole-2,3-dione ^(41)^

Compound **41** was prepared according to general procedure above by using 5, 7-dibromo-1H-indole-2, 3-dione and 4–(2-cyanophenyl)-benzyl bromide. Yellow power. Yield 90%; Mp 207–209 °C; ^1^H NMR (600 MHz, CDCl_3_)δ: 7.82 (d, J = 2.00 Hz, 1H), 7.75(dd, J = 7.83, 1.32 Hz, 1H), 7.72(d, J = 2.02 Hz, 1H), 7.63(td, J = 7.72, 1.40 Hz, 1H), 7.53(m, 2H), 7.48 (dd, J = 8.00, 1.15 Hz, 1H), 7.44 (td, J = 7.63, 1.19 Hz, 1H), 7.38 (d, J = 7.97 Hz, 2H), 5.47 (s, 2H); ^13^CNMR (151 MHz, CDCl_3_)δ: 181.24, 158.42, 146.65, 145.47, 144.73, 137.74, 136.45, 133.90, 133.01, 130.10, 129.35(2 C), 127.84, 127.70, 127.04(2 C), 121.52, 118.68, 117.41, 111.20, 105.35, 44.54; HRMS (ESI^+^)m/z: calcd for C_22_H_12_Br_2_N_2_O_2_ [M + Na]^+^ 518.9143, found 518.9095.

### Biology experiments

#### Enzyme inhibition assay

SARS-CoV-2 3CL^pro^ inhibition was performed via a SARS-CoV-2 3CL^pro^ inhibitor screening kit P0312 (Beyotime Biotechnology, Shanghai, China)^27^, which mainly used FRET methods and Dabcyl-KTSAVLQSGFRKM-E(Edans)-NH2 substrate. The ebselen and isatin derivatives were dissolved in 0.5% DMSO/H_2_O with different concentrations, respectively. The reaction mixture contained 92 μL of assay buffer, 5 μL of test compound solution, 1 μL of SARS-CoV-2 3CL^pro^, and 2 μL of the substrate in a black 96-well plate. After 10 min of incubation, the relative fluorescence unit (RFU) was read on a microplate reader (DU-730; Beckman Coulter, Brea, CA, USA) at the excitation of 340 nm and the emission of 490 nm. The calculation formula for inhibition rate is consistent with the previous report^27^.

#### Antiviral assay and cytotoxicity assay

The SARS-CoV-2 virus (strain IVCAS 6.7512) was provided by the National Virus Resource, Wuhan Institute of Virology, Chinese Academy of Sciences. To examine the anti-SARS-CoV-2 activity of test compound, plaque reduction assay was conducted. Briefly, Vero-E6 (Hefei Wanwu Biotechnology Co., Ltd, China) monolayers grown in 12 well plates were pre-treated by tested compound at various concentrations as 50, 15, 5, 1.5, 0.5, 0.15, 0.05 µM for 1h, followed by infection with SARS-CoV-2 (MOI of 0.0001) in the presence of test compound. DMSO and Ebselen were used as negative and positive controls respectively. After 1 h incubation, the medium was replaced with fresh MEM containing 1.25% Avicel and test compound, and the plates were incubated for another 48 h at 37 °C and 5% CO_2_. Then cells were fixed with 10% formalin and stained with 1% crystal violet to visualise plaques. The concentration required for the test compound to reduce the plaque formation of the virus by 50% (the 50% effective concentration [EC_50_]) was determined^24^. To estimate the cytotoxicity of test compound on VERO-E6 cells, Cell-Titer Glo^®^ luminescent cell viability assay (Promega) was performed according to the manufacturer’s instruction. The half of the cytotoxic concentration (CC_50_) values were calculated from the percentages of cells whose viability was inhibited by the test compound at various concentrations^24^.

#### Drug-likeness

Additionally, the drug candidate must reach its target in the body in sufficient concentration and stay there in a bioactive form long enough for the expected biologic events to occur. To check some properties related to this context, we take advantage of the SwissADME, that is a free web tool to evaluate pharmacokinetics, drug-likeness and medicinal chemistry friendliness of small molecules^25^. The Lipinski rule is used to distinguish between drug like and nondrug like molecules. This rule of 5 predicts high probability of success when there are less than 5 H-bond donors, 10 H-bond acceptors, the molecular weight (MWT) is less than 500 and the calculated Log P is less than 5^28^.

#### Reversibility of inhibition

A jump dilution assay is used to evaluate inhibition reversibility^29^. Compound **34** is pre-incubated for 1 h at 10 times of its IC_50_ with SARS-CoV-2 3CL^pro^ (100X). Then, the incubates are quickly diluted to 100-fold with substrate solution before measuring the fluorescence kinetics. Fluorescence intensity is measured 10 min and 2 h after dilution. The method of detecting the inhibitory rate of compound **34** in this assay refers to the instruction of SARS-CoV-2 3CL^pro^ inhibitor screening kit P0312 (Beyotime Biotechnology, Shanghai, China). In the same experiment, control pre-incubations of SARS-CoV-2 3CL^pro^ with compound **34** are performed (60 min) with just, as usual, a two-fold dilution with the substrate to obtain 10 × IC_50_ and 0.1 × IC_50_ final concentrations of compound **34**. All incubates are performed in triplicate.

#### Surface plasmon resonance (SPR) technique

For the surface plasmon resonance (SPR) assays^30^, 1.45 mg/mL SARS-CoV-2 3CL^pro^ was dialysed against 10 mM HEPES (pH 7.3), 1 mM DTT, and then diluted in 10 mM sodium acetate buffer (pH 5.0) to the final concentration of 100 μg/mL SARS-CoV-2 3CL^pro^. The assays were performed on a SPR system (Biacore T200; GE Healthcare, USA) at 25 °C. The SARS-CoV-2 3CL^pro^ was immobilised *via* the free amino groups using CM5 sensor chip coated with carboxymethylated dextran (Cytiva, USA). Before the covalent immobilisation of SARS-CoV-2 3CL^pro^, the chip surface was activated with 0.4 M 1-ethyl-3–(3-dimethylaminopropyl)-carboiimide hydro- chloride and 0.1 M N-hydroxy-succinimide. The injection of SARS-CoV-2 3CL^pro^ was adjusted to obtain 9000 response units of immobilised SARS-CoV-2 3CL^pro^ on the chip surface, and the uncoupled carboxymethyl groups were blocked with ethanolamine. The running buffer for the SPR assays was 10 mM HEPES (pH 7.4), 1 mM EDTA, 150 mM NaCl, 0.005% P20 and 5% DMSO. The samples were prepared by diluting compound **34** stock solutions in running buffer. The final concentration of DMSO in all of the compound **34** samples was 5%. Different concentrations of compound **34** (10, 3.3, 1, 0.33, 0.1, 0.033 µM) were injected over the chip surface for 60 s (250 s for ellagic acid). Dissociation of compound **34** from the chip surface was then monitored for 60 s (200 s for ellagic acid). Compound **34** that remained bound to the chip surface was removed after the dissociation phase by a 6 s pulse of 2 mM NaOH. The buffer flow rate was set at 30 μL/min for all of the phases of the SPR assays. Sensorgrams were corrected for the corresponding responses of flow–cell 1 without the immobilised SARS-CoV-2 3CL^pro^. The Biacore T200 evaluation software was used to determine the equilibrium dissociation constants (*K*_D_), by fitting the data to a steady-state affinity model or to two–state kinetics. All of the experiments were performed in duplicate.

#### Molecular docking studies

The small molecule was built using chemoffice (version 10.0) and converted to 3D structure from 2D structure. The crystal structure of SARS-CoV-2 3CL^pro^ (PDB ID 6LU7)^5^ was used as the template in the docking study. Water molecules and unwanted ligands were deleted, and polar hydrogens were added. Then the protein and small molecules were docked using Mol Plugin 4.16.0. The protocol generated 10 top hits for every small molecule, and random conformations were set to 10.

#### Molecular dynamics (MD) simulation

The simulation was conducted using the academic version of Desmond software package^31^. The system was solvated in a cubic box with periodic boundary conditions, using the TIP3P water model. Na^+^ and Cl^-^ ions were added to adjust the system to a concentration of 0.15 mol/l and to neutralise the total charge. The system was parameterised with the OPLS2005 force field, followed by a 1000 ps energy minimisation to eliminate unrealistic structures. Prior to the simulation, a system relaxation protocol was performed using the RESPA integrator with a 2fs time step. Additionally, a Nose-Hoover chain thermostat at 310 K with a 1 fs time step and a Martyna-Tobias-Klein barostat based on isotropic coupling at 1 atm (1.01325 bar) with a 2fs time step was employed for the relaxation protocol. Finally, a 100 ns molecular dynamics simulation was carried out under NPT conditions at 310 K and 1.01325 bar.

#### Statistical analysis

GraphPad Prism 8.0 software was used for the statistical analysis. All analytical experiments were repeated thrice, with values expressed as mean ± SD.

## CRediT authorship contribution statement

**Jian Song:** Writing – review & editing, Writing – original draft, Visualisation, Software, Methodology, Investigation, Formal analysis, Data curation. **Cai Shi:** Writing – review & editing, Writing – original draft, Visualisation, Software, Methodology, Investigation, Formal analysis, Data curation. **Boning Yang:** Writing – review & editing, Writing – original draft, Methodology, Investigation, Formal analysis. **Jingxiang Yang:** Writing–review & editing, Writing–original draft, Methodology, Investigation, Formal analysis. **Caifei Huang:** Writing–original draft, Writing–review & editing, Methodology, Investigation, Formal analysis. **Jiaqi Liu:** Writing–review & editing, Writing–original draft, Methodology, Investigation, Formal analysis. **Shuwen Wu:** Writing–original draft, Methodology, Investigation, Formal analysis, Conceptualisation. **Shuyuan Mo:** Writing–original draft, Visualisation, Software, Methodology, Investigation, Formal analysis, Conceptualisation. **Jian Yang:** Writing–review & editing, Writing–original draft, Supervision, Resources, Project administration, Methodology, Investigation, Funding acquisition, Conceptualisation. All authors critically reviewed the manuscript, contributed to its revision, approved the final version, and took full responsibility for the work.

## Supplementary Material

Supplementary Material2.docx

## Data Availability

The data presented in the current study are available from the corresponding author upon reasonable request.
