# Supplementary material for: Exploring novel isatin derivatives as SARS-CoV-2 3CLpro inhibitors
Source: J Enzyme Inhib Med Chem. 2026 Sep 10;41(1):2707859. doi: 10.1080/14756366.2026.2707859 (PMC13573511; doi:10.1080/14756366.2026.2707859)
Supplement: Supplementary file 1 [file IENZ_A_2707859_SM7680.docx]

**Supplementary material**

**Exploring novel isatin derivatives as SARS-CoV-2 Mpro inhibitors**

**Contents of Supplementary Information**

^1^H NMR, ^13^C NMR and MS of compounds **1** - **41**

HPLC of compound **34**

^1^H NMR, ^13^C NMR and MS of compounds **1**-**41**

1. *1-benzyl-indole-2,3-dione (****1****)*

2. *1-(4-methylbenzyl)-indole-2,3-dione (****2****)*

3. *1-(4-trifluoromethylbenzyl)-indole-2, 3-dione (3)*

4. *1-(2,4,6-trimethylbenzyl)-indole-2,3-dione (****4****)*

5. ***1-(4-isopropylbenzyl)-indole-2,3-dione (5)***

6. *1-(4-trifluoromethoxybenzyl)-indole-2,3-dione (6)*

7. *1-(4-nitrobenzyl)-indole-2,3-dione (****7****)*

8. *1-(4-cyanobenzyl)-indole-2,3-dione (****8****)*

9. *1-(4-carbomethoxybenzyl) -indole-2,3-dione (9)*

10. *1-(4-fluorobenzyl)-indole-2,3-dione (10)*

11. *1-(2, 3-difluorobenzyl)-indole-2, 3-dione (11)*

12. *1-(2,4,5-trifluorobenzyl)-indole-2,3-dione (12)*

13. *1-(4-chlorobenzyl)-indole-2,3-dione (13)*

*14. 1-(3, 4-dichlorobenzyl)-indole-2, 3-dione (14)*

15. *1-(2, 4-dichlorobenzyl)-indole-2, 3-dione(15)*

16. *1-(4-bromobenzyl)-indole-2,3-dione (16)*

17. *1-(8-quinolylmethyl)-indole-2, 3-dione (17)*

18. *1-(4-phenylbenzyl)-indole-2, 3-dione (****18****)*

19. *1-(1-carboxylic acid tert-butyl ester-indole-3-methyl)-indole-2, 3-dione (****19****)*

20. *1-allyl-indole-2, 3-dione (****20****)*

21. *1-(1-naphthylmethyl)-indole-2,3-dione (21)*

22. *1-(1-bromo-2-naphthylmethyl)-indole-2, 3-dione (22)*

23. *1-(1-bromo-4-naphthylmethyl) -indole-2, 3-dione (23)*

24. *1-(3-phenylbenzyl)-indole-2,3-dione (****24****)*

25. *1-(2-quinolylmethyl) -indole-2,3-dione (25)*

26. *1-(4-(2-cyanophenyl)-benzyl)-indole-2,3-dione (26)*

27. *1-(4-(2-carbomethoxyphenyl)-benzyl)-indole-2,3-dione (****27****)*

28. *5-fluoro-1-(4-(trifluoromethyl) benzyl)-indole-2, 3-dione (28)*

29. *5-chloro-1-(4-(trifluoromethyl)benzyl)-indole-2,3-dione (29)*

30. *5-bromo-1-(4-(trifluoromethyl)benzyl)-indole-2,3-dione (30)*

31. *4-chloro-1-(4-(trifluoromethyl)benzyl)-indole-2,3-dione (31)*

32. *6-chloro-1-(4-(trifluoromethyl)benzyl)-indole-2,3-dione (32)*

33. *4-iodo-1-(4-(trifluoromethyl) benzyl)-indole-2,3-dione (33)*

34. *5,7-dibromo-1-(4-(trifluoromethyl) benzyl)-indole-2, 3-dione (34)*

35. *4, 6-dichloro-1-(4-(trifluoromethyl) benzyl)-indole-2, 3-dione (35)*

36. *4-fluoro-5-chloro-1-(4-(trifluoromethyl) benzyl)-indole-2,3-dione (36)*

37*. 5,7-dibromo-1-(4-bromo-benzyl)-indole-2,3-dione (****37****)*

38. *5,7-dibromo-1-(4-phenylbenzyl)-indole-2,3-dione (38)*

39. *5,7-dibromo-1-(4-nitro-benzyl)-indole-2,3-dione (39)*

40. *5,7-dibromo-1-(4-cyano-benzyl)-indole-2,3-dione (****40****)*

41. *5,7-dibromo-1-(4-(2-cyanophenyl)-benzyl)-indole-2,3-dione (****41****)*

HPLC of compound **34**

| Pk # | Retentn Time | Area | Area Percent | Asymmty |
| --- | --- | --- | --- | --- |
| 1 | 5.73 | 1778.518 | 0.18 | 0.00 |
| 2 | 5.83 | 631.056 | 0.07 | 0.00 |
| 3 | 7.21 | 966060.970 | 99.75 | 0.00 |
| Totals |  | 968470.544 | 100 |  |
